# Wise Roles and Future Visionary Endeavors of Current Emperor: Advancing Dynamic Methods for Longitudinal Microbiome Meta‐Omics Data in Personalized and Precision Medicine

**DOI:** 10.1002/advs.202400458

**Published:** 2024-11-13

**Authors:** Vera‐Khlara S. Oh, Robert W. Li

**Affiliations:** ^1^ Big Biomedical Data Integration and Statistical Analysis (DIANA) Research Center Department of Data Science College of Natural Sciences Jeju National University Jeju City Jeju Do 63243 South Korea; ^2^ United States Department of Agriculture Agricultural Research Service Animal Genomics and Improvement Laboratory Beltsville MD 20705 USA

**Keywords:** artificial intelligence, dynamic methods, longitudinal meta multi‐omics, microbiome, personalized/precision medicine

## Abstract

Understanding the etiological complexity of diseases requires identifying biomarkers longitudinally associated with specific phenotypes. Advanced sequencing tools generate dynamic microbiome data, providing insights into microbial community functions and their impact on health. This review aims to explore the current roles and future visionary endeavors of dynamic methods for integrating longitudinal microbiome multi‐omics data in personalized and precision medicine. This work seeks to synthesize existing research, propose best practices, and highlight innovative techniques. The development and application of advanced dynamic methods, including the unified analytical frameworks and deep learning tools in artificial intelligence, are critically examined. Aggregating data on microbes, metabolites, genes, and other entities offers profound insights into the interactions among microorganisms, host physiology, and external stimuli. Despite progress, the absence of gold standards for validating analytical protocols and data resources of various longitudinal multi‐omics studies remains a significant challenge. The interdependence of workflow steps critically affects overall outcomes. This work provides a comprehensive roadmap for best practices, addressing current challenges with advanced dynamic methods. The review underscores the biological effects of clinical, experimental, and analytical protocol settings on outcomes. Establishing consensus on dynamic microbiome inter‐studies and advancing reliable analytical protocols are pivotal for the future of personalized and precision medicine.

## Graphical abstract

The authors summarize the most recent advances in handling longitudinal omics data in microbiome studies and precision medicine. They highlight limitations and constraints in the theory and practice in the current research. The authors also advocate the development of artificial intelligence (AI)‐centric pipelines for integrated multi‐omics analyses to understand dynamic processes in microbial ecology and microbiota engineering.

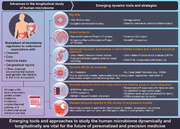

## 1 Introduction

Numerous studies on the human microbiome, including those on gut flora using 16S rRNA marker gene surveys and whole genome shotgun (WGS) metagenomic sequencing, have been successfully conducted.^[^ ^1^, ^2^, ^3^, ^4^, ^5^, ^6^, ^7^, ^8^ ^]^ Most of these studies aimed to characterize the interplay between microbial communities and phenotypes, such as human diseases, therapeutic effects, and other interventional factors, especially diet.^[^ ^5^, ^9^, ^10^, ^11^, ^12^, ^13^, ^14^, ^15^, ^16^, ^17^, ^18^ ^]^

Due to the limited value of cross‐sectional data, repeated and longitudinal large‐scale microbiome data in cohort studies are becoming increasingly popular for identifying phenotypic biomarkers.^[^ ^19^, ^20^, ^21^, ^22^, ^23^, ^24^, ^25^, ^26^, ^27^, ^28^, ^29^, ^30^, ^31^, ^32^ ^]^ Longitudinally measured human microbial samples in response to stimuli, such as therapies, reveal temporal changes in operational taxonomic abundance, structural composition, and enriched functionality.^[^ ^15^, ^23^, ^29^, ^30^, ^33^, ^34^, ^35^, ^36^ ^]^ Integrating dynamic data through meta‐strategies across intra‐ and inter‐platforms (e.g., multi‐omics data) has become feasible in the field.^[^ ^24^, ^37^, ^38^, ^39^, ^40^, ^41^ ^]^ However, a major challenge in these meta‐analyses is handling data contamination from systematic biases when merging multiple heterogeneous datasets, alongside addressing the biological impact of protocol choices in clinical, experimental, or analytical settings on final outcomes in mainstream analyses.^[^ ^25^, ^42^, ^43^, ^44^, ^45^ ^]^ These issues can lead to spurious results in subsequent analyses, including univariate longitudinal differential abundance tests, functional enrichments, causal inferences of directed network modules, and predictive models for monitoring patient‐based diagnostic, prognostics, and drug treatment effects.^[^ ^46^, ^47^, ^48^, ^49^, ^50^ ^]^ Additionally, the lack of publicly available large‐scale longitudinal or multi‐omics datasets, versatile statistical and computational frameworks capable of handling complex microbiome data with bigger dimensions, and systematic comparative studies to validate the interactive robustness and reproducibility across different studies pose significant bottlenecks.^[^ ^51^, ^52^, ^53^, ^54^, ^55^, ^56^, ^57^ ^]^

To effectively analyze dynamic spectral changes in dysbiosis perturbed by external factors, dynamic methods must explicitly address intrinsic data‐driven characteristics of microbiome counts, such as compositionality, excessive zeros and missing values in sparsity, sample size imbalance between different groups, intra‐ and inter‐individual heterogeneity in individuals, and systematic artifacts in data integration processes.^[^ ^20^, ^58^, ^59^, ^60^, ^61^, ^62^, ^63^, ^64^, ^65^, ^66^, ^67^, ^68^, ^69^, ^70^, ^71^, ^72^ ^]^

In this comprehensive review, we focus on discussing current challenges and future research directions, particularly concerning advanced dynamic methods applied to understand dynamic biomolecular processes in microbiome‐based personalized and precision medicine. These dynamic methods play a crucial role at various stages of mainstream analyses within the workflow, including feature‐by‐feature or feature‐to‐feature dynamic approaches based on the quantification of microbiome counts or normalized/transformed data. The tasks are typically managed by biostatisticians, bioinformaticians, and developers. In this study, we aimed to aid careful navigation of the critical issues related to the state‐of‐the‐art dynamic methods, thereby reducing misleading results due to the biological impact of data management at pre‐steps onto the final outcomes. We propose data‐driven validation procedures to ensure simple and robust analytical protocols, which can promote consistent conclusions across studies and enhance reproducibility and reliability in inter‐study comparisons beyond specific methodological or analytical pipeline results. Our goal is to provide a comprehensive workflow roadmap illustrating the application of dynamic methods for future studies integrating longitudinal multi‐omics microbiome data in biomedical research (**COVER PICTURE**). In the following sections, we discuss the evolution of microbiome studies toward personalized and precision medicines in Section 1. We introduce two major types of microbiome sequencing technologies in Sections 1.1 and explore the roles of dynamic longitudinal microbiome studies in Section 1.2. Section 2 covers national and international large microbiome consortia, highlighting their potential impact on human diseases and public health. Sections 3 and 4 delve into advanced dynamic approaches, examining feature‐by‐feature and feature‐to‐feature methodologies, respectively, along with their rationales, advantages, limitations, and future opportunities. We underscore the ultimate goals of dynamic multi‐omics studies as the frontiers in Section 5. In this section, we emphasize the overarching goals of dynamic multi‐omics studies as frontiers in microbiome research. Section 6 focuses on recent microbiome studies that analyze disease temporal spectra. Finally, we conclude with a discussion and closing remarks, proposing optimal guidelines for comprehensive analytical protocols with the advanced dynamic methods as best practices in modern medical research, particularly in personalized and precision medicine.

## 2 Evolution of Microbiome Studies

The microbiome encompasses the entire habitat, including microorganisms (bacteria, archaea, eukaryotes, and viruses), their genomes, and the surrounding environmental conditions.^[^ ^73^ ^]^ The gut microbiome is crucial for host development, physiology, and pathology, and it is linked to various human diseases such as autism, autoimmune diseases (e.g., multiple sclerosis, rheumatoid arthritis, colitis), brain disorders (e.g., Alzheimer's, Parkinson's), tumors, and yellow fever.^[^ ^35^, ^74^, ^75^, ^76^, ^77^, ^78^, ^79^, ^80^, ^81^, ^82^, ^83^, ^84^, ^85^ ^]^

Consequently, rapid detection of pathogens (bacteria, viruses, fungi, and parasites), virulence factors, and antimicrobial resistance genes, along with the assessment of patient microbiome responses, should be integrated into routine clinical diagnostics. Additionally, understanding gut microbiome responses is vital for evaluating drug interactions.

Recent studies highlight the microbiome's importance in precision medicine.^[^ ^14^, ^15^, ^75^, ^86^, ^87^ ^]^ Researchers have aimed to establish microbiome‐encoded disease phenotypes.^[^ ^2^, ^6^, ^19^, ^21^, ^23^, ^24^, ^30^, ^79^, ^81^, ^88^, ^89^, ^90^, ^91^, ^92^, ^93^, ^94^, ^95^ ^]^ The gut microbiome is essential for host immunity development,^[^ ^77^, ^78^, ^80^, ^96^, ^97^ ^]^ and variations in gut microbiota composition impact individual vaccination responses.^[^ ^96^ ^]^ Thus, tools for precise gut microbiome regulation can improve vaccine efficacy. Additionally, studies indicate that microbial signatures can predict treatment outcomes.^[^ ^12^, ^23^, ^26^, ^29^, ^30^, ^35^, ^48^, ^75^, ^76^, ^77^, ^81^, ^88^, ^97^, ^98^, ^99^, ^100^, ^101^ ^]^ Evidence supports precision microbiome manipulation using specific substances for desired metabolic outputs,^[^ ^102^ ^]^ suggesting that precise gut microbiome editing could be a therapeutic option. For instance, dysbiotic Enterobacteriaceae expansion during gut inflammation can be prevented by selectively inhibiting molybdenum‐cofactor‐dependent microbial respiratory pathways with tungstate.^[^ ^75^ ^]^

### 2.1 Introduction to Microbiome Sequencing Technologies

The gut microbiome is a complex, dynamic open system that frequently exchanges materials and energy with its environment. Microbial abundance within a microbiome varies temporally or due to environmental changes or disturbances.^[^ ^18^, ^103^, ^104^, ^105^, ^106^, ^107^ ^]^ This complexity and functional significance of the microbiome necessitates advanced research methods and bioinformatics algorithms for comprehensive studies using culture‐independent approaches.

#### 2.1.1 rRNA Marker Gene Surveys

To this end, marker gene‐based microbiome profiling is widely employed in microbiome.^[^ ^1^, ^108^, ^109^, ^110^, ^111^ ^]^ Among these, the 16S rRNA gene, a small subunit (SSU) of rRNA, is commonly used for identifying and classifying bacteria and archaea at genus and species levels. For fungi and other eukaryotes, the 18S rRNA gene and internal transcribed spacer (ITS) sequences serve as key phylogenetic markers.^[^ ^112^, ^113^, ^114^, ^115^ ^]^ The hypervariable regions in these marker genes allow for the differentiation of closely related species.^[^ ^116^, ^117^ ^]^ Additionally, the highly variable ITS region, located between the 18S and 5.8S rRNA genes, is instrumental in identifying various fungi and capturing microbial diversity.^[^ ^118^, ^119^ ^]^ Decades of collective efforts have led to the creation of multiple SSU reference databases, such as Greengenes,^[^ ^109^, ^120^ ^]^ Silva,^[^ ^121^ ^]^ RDP,^[^ ^122^ ^]^ and UNITE.^[^ ^123^ ^]^ These databases facilitate marker gene‐based microbial surveys in specific habitats or environmental samples and the integration of these reference databases with advanced bioinformatics tools has significantly enhanced the accuracy and efficiency of microbiome profiling.^[^ ^124^ ^]^

#### 2.1.2 Whole Genome Shotgun Metagenomic Sequencing

The rapid advancement of ultralow‐cost, high‐throughput parallel sequencing technologies, and bioinformatics tools has driven WGS‐based computational metagenomics. This approach provides an unbiased, comprehensive analysis of the functional and metabolic potential of the microbiome.^[^ ^1^, ^3^, ^40^, ^125^, ^126^, ^127^, ^128^, ^129^, ^130^, ^131^, ^132^, ^133^ ^]^ A key goal of WGS‐based metagenomics sequencing is the swift identification and characterization of metagenome‐derived genomes (MAGs) from the unculturable fraction of the microbiome. WGS‐based metagenomic data are voluminous, often including millions of paired‐end reads per sample and trillions of base (Tb) pairs of raw data per study from various platforms. These platforms generate short reads (e.g., Illumina), long reads (e.g., Nanopore), and hybrid reads. Large WGS projects may involve multiple centers or institutions, contributing to data heterogeneity. Furthermore, species abundance in metagenome samples varies significantly, posing unique data analysis challenges.^[^ ^1^, ^126^, ^127^, ^128^, ^129^, ^130^, ^133^, ^134^, ^135^ ^]^

### 2.2 Dynamic Longitudinal Microbiome Studies

**Figure 1:**
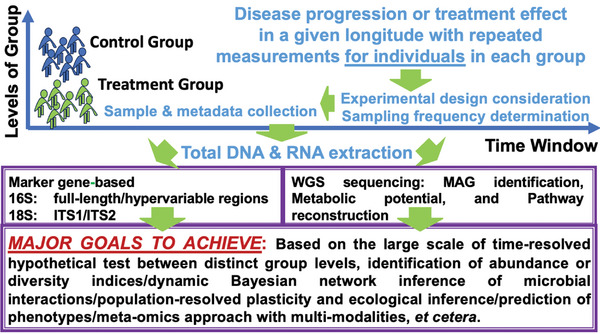
Schematic illustration of microbiome‐based cohort study over time with external factors at distinct levels in groups. Abbreviations^a^: internal transcribed spacer (ITS1/ITS2); Whole genome shotgun (WGS); metagenome‐derived genomes (MAG); ^a^The ITS1 region ranges from 50 to 350 bp and is located between the 18S rRNA and 5.8S rRNA genes. The ITS2 region is of 50–650 bp‐long and is located between the 5.8S rRNA and LSU (28S) rRNA genes. For the further details, please refer to as.^[^ ^114^ ^]^

Many microbiome studies are cross‐sectional, effectively capturing MAGs and providing insights into the metabolic potential of microbial communities.^[^ ^136^, ^137^, ^138^, ^139^ ^]^ However, they are static and limited in assessing the microbiome's temporal dynamics.^[^ ^140^ ^]^ They cannot fully observe inter‐ and intra‐individual variations due to genetic and lifestyle heterogeneity among human subjects.^[^ ^24^, ^81^, ^141^, ^142^ ^]^ These variations and background noise obscure not all, but the most significant differences, complicating clinical correlation inference. Time‐series microbiome studies address these limitations, enhancing understanding of individual variations and improving clinical trial interventions (**Figure** **1** and Supplementary Table S1, Supporting Information).^[^ ^19^, ^20^, ^21^, ^22^, ^23^, ^24^, ^26^, ^27^, ^28^, ^81^, ^89^, ^138^ ^]^ Longitudinal studies offer novel insights into the dynamic behavior of the microbiome and help infer causality in microbiome‐disease associations, though tools addressing common issues like subject dropout and irregular sampling are still needed.^[^ ^141^, ^143^, ^144^, ^145^ ^]^ Recent advancements have introduced promising tools tailored for analyzing time‐series microbiome data. For instance, the R package “splinectomeR” uses smoothing splines for hypothesis testing in longitudinal studies, allowing comparisons of parameters such as alpha diversity.^[^ ^143^ ^]^ Traditional algorithms often treat time‐series data as static snapshots, overlooking temporal dependencies and nonlinear species relationships.^[^ ^56^, ^146^ ^]^ Applying dynamic systems theory, Berry and Widder (2014)^[^ ^147^ ^]^ utilized generalized Lotka–Volterra (gLV) models to infer dynamic network interactions, emphasizing keystone species’ roles. The Learning Interactions from Microbial Time Series (LIMITS) approach identifies interaction networks dominated by distinct keystone species.^[^ ^148^ ^]^ Additionally, Alshawaqfeh et al.’s algorithm demonstrates robust performance against model uncertainty with over 75% accuracy, outperforming several algorithms, including LIMITS, in inferring microbial interactions.^[^ ^56^, ^146^, ^148^ ^]^

This review discusses recent advancements in dynamic tools for analyzing longitudinal time‐series microbiome coupled with multi‐omics data for the further details in section 3 through 5. It emphasizes the importance of longitudinal microbiome studies for the better characterization of the variety of dynamic processes in personalized and precision medicine, addressing the limitations and potential pitfalls of advanced dynamic methods and algorithms, including machine learning (ML) and deep learning (DL) approaches in artificial intelligence (AI).

## 3 Large Microbiome Consortia and Their Impact

**Figure 2:**
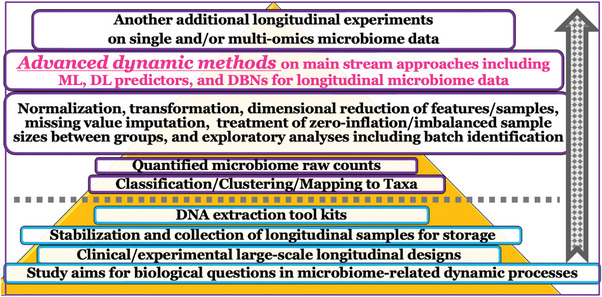
Effects of pre‐steps on the results of mainstream analyses with dynamic methods within the entire bottom‐up pyramid workflow for longitudinal microbiome data.

Large national and international consortia, involving multiple leading researchers and their laboratories, aim to deepen the systematic understanding of microbiome systems related to ecology and human health.^[^ ^10^, ^24^, ^50^, ^81^, ^107^ ^]^ These consortia are supported by substantial research funding and valuable data resources, which the investigators pursue their research goals effectively. To achieve future research objectives, establishing standardized clinical, experimental, and analytical protocols across the entire workflow and ensuring accurate characterization of dynamic processes in various longitudinal microbiome data is crucial (**Figure** **2**).^[^ ^46^, ^50^, ^51^, ^52^, ^53^, ^54^, ^55^, ^149^ ^]^ To avoid misleading results and achieve universal conclusions on microbiome disease associations, comprehensive comparative evaluations should be conducted to assess reproducibility, robustness, and validity, while controlling biological data contamination throughout clinical, experimental, and analytical processes.^[^ ^51^, ^53^, ^150^ ^]^ This section reviews key large‐consortia projects in the microbial field, emphasizing their contributions to advancing our understanding of microbiome ecology and its implications for human health.

### 3.1 Human Microbiome Project (HMP Phase I)

The study aimed to characterize the normal microbiota in western populations from various body habitats, including the gut, skin, and vagina, using extensive sampling and both 16S rRNA marker surveys and WGS metagenomics data.^[^ ^10^ ^]^ It provided insights into the diversity of healthy microbiomes and their variations due to dysbiosis in various human disorders, as highlighted in epidemiological studies.^[^ ^2^, ^32^, ^34^, ^76^, ^77^, ^81^, ^151^, ^152^ ^]^ The project revealed significant intra‐ and inter‐subject variability, with differences in immune responses to pathogens and environmental factors at various levels, including taxa, strains, genes, metabolites, functionalities, and synergistic interactions.^[^ ^4^, ^20^, ^30^, ^33^, ^34^, ^35^, ^36^, ^37^, ^41^, ^74^, ^76^, ^96^, ^107^, ^151^, ^153^, ^154^ ^]^ It emphasized the importance of accounting for intra‐ and inter‐subject heterogeneity in microbiome data to ensure accurate long‐ and short‐term effects in personalized and precision diagnostics and therapies.^[^ ^12^, ^36^, ^48^, ^75^, ^87^, ^98^, ^100^, ^102^, ^153^, ^155^, ^156^, ^157^ ^]^

### 3.2 Integrative Human Microbiome Project (iHMP, Phase II of HMP)

Most molecular mechanisms influenced by shifts in human microbiome ecosystems under perturbed stimuli, such as targeted therapies and diseases, are dynamic.^[^ ^13^, ^24^, ^27^, ^28^, ^31^, ^34^, ^81^, ^158^ ^]^ Consequently, phase II of HMP utilized longitudinal microbiome cohort studies with deep sequencing coverage and dense sampling.^[^ ^24^ ^]^ Analyses based solely on single‐omics, microbiome data are suboptimal. Therefore, phase II aimed to define human‐associated microbiota across various disease states using multi‐omics data, including transcriptomics, proteomics, metabolomics, genetic risk factors, and clinical information.^[^ ^24^, ^35^, ^41^, ^48^, ^79^, ^106^, ^159^ ^]^ However, methods to handle the massive, heterogeneous multi‐omics data remain elusive.

### 3.3 American Gut Project

A substantial gut consortium has been established in the meta‐strategy of microbiome and metabolomics data, utilizing populations from the USA, UK, and Australia.^[^ ^107^ ^]^ This initiative aims to identify metabolic compounds and their functional roles derived from interactions among microbes, host physiology, and external factors in meta‐multi‐omics strategies. Sub‐grouped individual samples, categorized based on environmental factors such as mental illness indicators and antibiotic use during surgery, were included.^[^ ^107^ ^]^ Metrics also considered lifestyle/dietary types and surgical effects, showing significant differences in alpha diversity.^[^ ^107^ ^]^ Notably, the gut flora constitutes a vast ecosystem with trillions of microorganisms interacting with the brain, heart, and liver axis.^[^ ^36^, ^76^ ^]^ Beyond single‐layered approaches with longitudinal microbiome data, well‐matched multi‐omics datasets from genes, metabolites, and proteins of microbial communities provide additional layers of information on transcriptional genes, metabolic enzymes, and peptides. These data elucidate the roles of microbes in disease progression models and their therapeutic effects, offering more comprehensive and confirmatory strategies. Acknowledging the value of multi‐omics longitudinal microbiome data, standardized experimental, clinical, and analytical protocols across omics levels should be developed.^[^ ^24^, ^154^ ^]^ Enhanced dynamic multi‐omics methods in future studies should be implemented in publicly accessible and communicable frameworks for researchers, including clinicians, biologists, analysts, and developers in the microbiome community.

### 3.4 MetaHIT Consortium Funded by Europe (MetaHIT Project)

A revised curation of reference genes for the human gut microbiome, encompassing bacteria and archaea, has been proposed to enhance meta‐strategies by updating previously annotated gene catalogs.^[^ ^106^, ^134^ ^]^ This catalog can be improved by stringent mathematical assessments in systematic comparisons, accounting for individual variabilities like geographical and environmental factors, particularly regarding core essential and rare genes with low detection rates due to sequencing depth.

Studies on HMP,^[^ ^10^ ^]^ iHMP,^[^ ^24^ ^]^ AGP,^[^ ^107^ ^]^ and the metaHIT Consortium^[^ ^106^, ^134^ ^]^ represent significant investments.^[^ ^10^, ^24^, ^107^ ^]^ These initiatives, benefiting from advancements in microbiome research, position US and global leaders as role models for longitudinal microbiome cohort studies in personalized and precision medicine. Future studies ***should be well‐balanced*** (balanced sample sizes across all factors in cohorts), ***well‐defined*** (clearly defined target populations regarding disease phenotypes, stages, race, region, age, and other covariates), ***well‐designed*** (large‐scale cohorts with adequate power and effect sizes), ***well‐followed*** (minimal data loss during follow‐up), ***well‐controlled*** (control for confounding covariates affecting main phenotypes), and ***well‐integrated*** (incorporating metagenomics, metaproteomics, metatranscriptomics, metabolomics, and health records, as isolated findings may not be universal). This approach aims to address microbiological questions related to biomolecular processes in human diseases and treatments and tackles challenges in clinical, experimental, and analytical protocols for longitudinal microbiome multi‐omics data in personalized and precision medicine.

## 4 Advanced Univariate Dynamic Approaches

**Figure 3:**
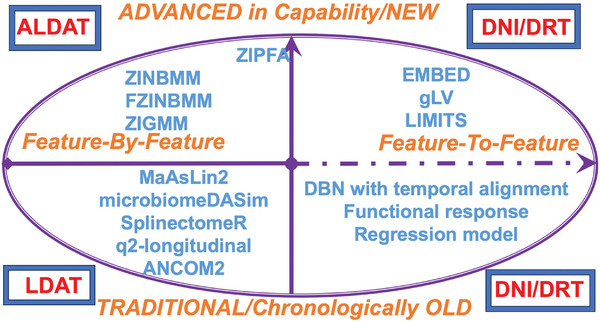
Advanced dynamic methods for the characterization of temporal dynamics within the perspective of feature‐by(‐to‐)‐feature strategies. Abbreviations^a^: ANCOM2 (Analysis of Composition of Microbiomes); ALDAT (Advanced Longitudinal Differential Abundance Test); DBN (Dynamic Bayesian Network); DNI (Dynamic Network Inference); DRT (Dimensional Reduction Technique); EMBED (Essential MicroBiomeE Dynamics); FZINBMM (Fast Zero‐Inflated Negative Binomial Mixed Model); gLV (Generalized Lotka‐Volterra); LDAT (Longitudinal Differential Abundance Test); LIMITS (Learning Interactions from Microbial Time Series); ZIGMM (Zero‐Inflated Gaussian Mixture Model); ZINBMM (Zero‐Inflated Negative Binomial Mixed Model); ZIPFA (Zero‐Inflated Poisson Factor Analysis).; ^a^Those inside the purple oval are discussed in the present review.

Dynamic microbiome data‐oriented methods can identify temporal changes in longitudinal differential abundance tests, yielding statistical significance via p‐value or posterior probability.^[^ ^25^, ^37^, ^67^, ^69^, ^70^, ^131^, ^143^, ^159^, ^160^, ^161^ ^]^ Initial univariate dynamic methods are detailed in our previous study.^[^ ^25^ ^]^ Methodological issues needing resolution include microbiome count properties, over/under dispersion, compositionality, zero‐inflation, and sparsity of low‐abundant features.^[^ ^22^, ^58^, ^59^, ^61^, ^63^, ^66^, ^67^, ^131^, ^162^, ^163^ ^]^ Dynamic methods must address missing values in repeatedly measured samples.^[^ ^62^, ^71^, ^164^ ^]^ Additionally, flexibility to incorporate clinical nuisance factors and data contamination from aggregation and preprocessing remain challenges.^[^ ^25^, ^42^, ^43^, ^44^, ^54^, ^165^, ^166^, ^167^, ^168^, ^169^, ^170^, ^171^ ^]^ Accurate estimation of intra/inter heterogeneity in longitudinal designs is complex.^[^ ^44^ ^]^ Systematic artifacts persist despite tailored normalization techniques.^[^ ^25^, ^43^, ^44^, ^168^, ^169^, ^172^, ^173^ ^]^ Enhanced dynamic methods with the current challenges are introduced in **Figure** **3** and Table S2, Supporting Information.

### 4.1 Paulson et al. (Zero‐Inflated Gaussian Mixed Model: ZIGMM)

This model is based on **the mixture of zero‐inflated Gaussian distribution** of mean group differences at each taxonomic feature, using log‐transformed data with cumulative sum scaling normalization in the metagenomeSeq R package.^[^ ^69^, ^70^ ^]^ This study represents the first attempt to address zero inflation in microbiome counts. The performance of this method was compared to static methods like DESeq2^[^ ^172^ ^]^ and edgeR.^[^ ^173^, ^174^ ^]^ The findings suggest that extensive systematic comparative studies, including other zero‐inflated models, would be advantageous for future research.^[^ ^58^, ^59^, ^61^, ^64^, ^65^, ^66^, ^67^, ^68^, ^163^, ^175^, ^176^ ^]^ Further debates should be also discussed if the log‐transformed data is preferable over inherent microbiome counts for dynamic methods.^[^ ^177^, ^178^ ^]^ Validation tasks should assess the performance of multiple methods, focusing on the biological impact of preprocessing steps on the final results of the dynamic methods (discussed in details in later sections). Additionally, this dynamic method does not account for other covariates, such as batch sources, necessary for diverse meta‐longitudinal time‐series microbiome data across both intra‐ and inter‐platforms.

### 4.2 Zhang et al. (Zero‐Inflated Negative Binomial Mixed Model: ZINBMM → Fast ZINBMM: FZINBMM)

Zhang et al.^[^ ^58^ ^]^ developed a zero‐inflated model with efficient computation time in R, crucial for microbiome longitudinal data with moderate to large sample sizes and extended time points.^[^ ^58^, ^59^, ^60^, ^66^, ^163^ ^]^ This model surpassed the ZIGMM and other common methods in detection power and true discovery rates across various simulations. However, like ZIGMM,^[^ ^70^ ^]^ FZINBMM does not fully accommodate the integration of other nuisance factors, such as systematic batch artifacts in multiple longitudinal microbiome datasets.^[^ ^25^, ^42^, ^45^, ^54^, ^165^, ^166^, ^167^, ^168^, ^169^, ^170^ ^]^

### 4.3 Xu et al. (ZIPFA)

Unlike ZIGMM^[^ ^70^ ^]^ and FZINBMM,^[^ ^58^ ^]^ this approach utilized a feature‐to‐feature dynamic method, named with zero‐inflated Poisson factor model.^[^ ^61^ ^]^ ZIPFA addressed issues including (i) varying library size factors; (ii) extra biological variation and systematic errors; (iii) excessive zero counts; (iv) overdispersion in zero counts; and (v) a fixed probability for true biological zeros, indicating the absence of taxa in samples per feature. The method used a taxon‐specific zero‐inflated Poisson model inferred by expectation and maximization, linking zero probabilities and Poisson rates in a logistic model. ZIPFA preserved the original count data structure without transformation. This is crucial as a recent large‐scale benchmarking study found bulk RNA‐seq‐specific count methods performed better for static microbiome data than methods requiring count transformation like metagenomeSeq.^[^ ^177^ ^]^ However, ZIPFA was initially used for single‐layer longitudinal microbiome data, not for more complex integrated data types with multiple time‐course datasets where data contamination could be a prevalent phenomenon.^[^ ^25^, ^42^, ^45^, ^54^, ^165^, ^166^, ^167^, ^168^, ^169^, ^170^ ^]^

A recent comparative study on zero‐inflated Poisson, Negative Binomial (NB), and hurdle models^[^ ^64^ ^]^ indicated that zero‐inflated methods outperform when zero inflation is significant, despite computational complexities like non‐convergence and local maxima, compared to hurdle models (Supplementary Table S2, Supporting Information). However, these evaluations were based on static microbiome data and simulations.

### 4.4 MMUPHin Incorporated with MaAsLin2

This unified dynamic strategy defines temporal associations between dysbiosis and disease risk indicators.^[^ ^44^ ^]^ Most existing dynamic methods^[^ ^25^, ^42^, ^43^, ^44^, ^45^, ^54^, ^70^, ^165^, ^166^, ^167^, ^168^, ^169^, ^170^, ^179^ ^]^ utilize separate step‐wise analytical pipelines, requiring users to manually handle preprocessing. Different preprocessing tool choices by users can lead to varying results, affecting reproducibility across studies. In contrast, this study developed a unified dynamic analytical protocol that integrates multiple steps^[^ ^44^ ^]^: (1) data correction preprocessing, such as batch adjustments via Combat with a zero‐inflation factor, (2) longitudinal differential abundance testing, and (3) unsupervised main‐stream analyses. The performance of MMUPHin was evaluated using various batch adjustments, longitudinal differential abundance tests, unsupervised clustering in discrete and continuous structure discovery, demonstrating significant superiority.^[^ ^44^ ^]^ However, systematic validation is needed to verify its superior performance by including recently developed methods like Harman/percentile normalization/BDMMA^[^ ^42^, ^43^, ^169^ ^]^ and zero‐inflated negative binomial approaches.

A unified dynamic analytical protocol integrating NBMM and Hazard function in survival analysis was developed to estimate posterior hazard ratios and event‐free probabilities, identifying significant changes in microbial features related to disease onset.^[^ ^180^ ^]^ However, it needs to address current challenges in longitudinal compositional counts, such as zero‐inflation, missing values in sparsity, and intra‐/inter‐heterogeneity. Additionally, this method should incorporate batch factors to handle various metadata, including integration strategies for longitudinal microbiome cohort studies from multiple clinical centers (**See impact of pre‐steps of analytical workflows in** Figure 2 **and BM methods in** Table S1, Supporting Information).

**In summary,** **Section** **3** highlights that advanced dynamic approaches in feature‐by‐feature univariate longitudinal differential abundance tests often assume feature independence, overlooking the co‐abundance of features that may indicate co‐functional associations^[^ ^24^, ^33^, ^39^, ^57^, ^71^, ^132^, ^144^, ^154^, ^161^, ^164^, ^171^, ^181^, ^182^, ^183^, ^184^, ^185^, ^186^ ^]^ in dynamic processes. Typically, temporal changes at each feature are inferred through a uniform model with multiple corrections rather than best‐fit model selection strategies^[^ ^58^, ^59^, ^66^ ^]^ and feature selection schemes to identify key contributors.^[^ ^153^, ^187^ ^]^ Although univariate longitudinal differential analysis with dynamic methods is essential to identify the significantly temporally varying changes of features in longitudinal clinical and experimental designs and explore their roles in functional enrichment analyses, we discuss advanced dynamic ML and DL methods in AI, causal inferential network modules with DBNs and supervised predictors, as feature‐to‐feature approaches in the next section.

## 5 Advanced Dynamic ML and DL Methods in AI

**Figure 4:**
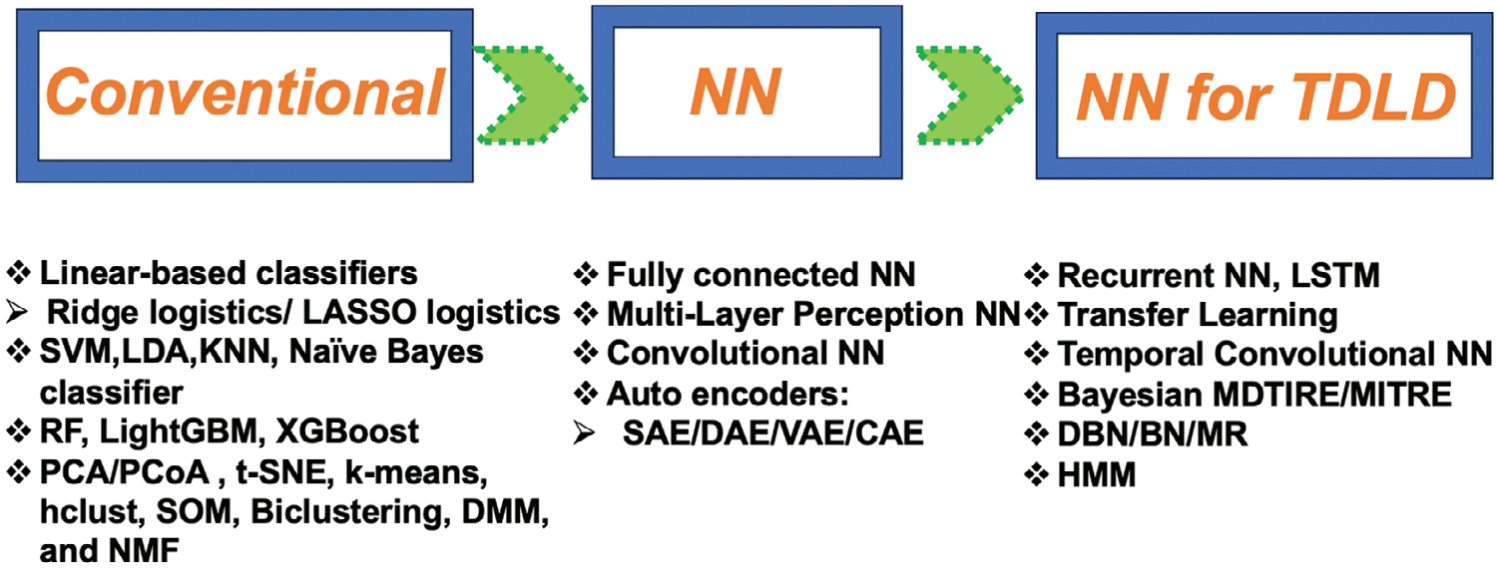
Simple supervised linear‐based classifiers and enhanced ML and DL methods in AI tools for co‐abundance clustering, prediction/classification, and dynamic network inference tools for longitudinal microbiome data. Abbreviations: NN (Neural Networks); AE (Autoencoder); BN (Bayesian Network); BNN (Bayesian Neural Network); CAE (Convolutional Autoencoder); DAE (Deep Autoencoder); DBN (Dynamic Bayesian Network); DMM (Dirichlet Multinomial Mixture); hclust (Hierarchical Clustering); HMM (Hidden Markov Model); KNN (K‐Nearest Neighbor); LDA (Linear Discriminant Analysis); LightGBM (Light Gradient Boosting Machine); LSTM (Long Short Term Memory); MDTIRE (Microbiome Differentiable Interpretable Temporal Rule Engine); MITRE (Microbiome Interpretable Temporal Rule Engine); MR (Mendelian Randomization); NMF (Non‐negative Matrix Factorization); PCA (Principle Component Analysis); PCoA (Principle Coordinate Analysis); RF (Random Forest); SOM (Self‐Organizing Map); SAE (Shallow Autoencoder); SVM (Support Vector Machine); TDLD (Temporally Dynamical Longitudinal Data); tSNE (t‐distributed Stochastic Neighbor Embedding); VAE (Variational Autoencoder); XGBoost (Extreme Boosting Decision Tree).

Dynamic ML and DL tools in AI have been employed for longitudinal microbiome data in personalized and precision medicine^[^ ^153^, ^155^, ^183^, ^186^, ^188^, ^189^, ^190^, ^191^, ^192^, ^193^, ^194^ ^]^ to identify pathological roles for initial disease diagnosis, stratification, monitoring critical patient care, and individualized therapies for drug response efficacy (**Figure** **4** and Table S3, Supporting Information)^[^ ^48^, ^101^ ^]^

### 5.1 Predictor 1 of ML and DL in AI

Metwally et al. advanced ML applications in AI for temporally dynamic microbiome data in cohort studies, using publicly available longitudinal microbiome data (**DIABIMMUNE**) on food allergies across three regions.^[^ ^195^ ^]^ It aimed to assess the predictive accuracy of various temporal dynamic‐specific classifiers, and revealed that long short‐term memory (LSTM), a type of recurrent neural network (RNN), performed best due to its ability to account for temporal dependencies. The predictive results were validated through different feature selection schemes,^[^ ^95^, ^195^, ^196^, ^197^ ^]^ including a sparse Auto Encoder (AE) selection criterion. Compared to traditional methods like Random Forest (RF), the LSTM combined with sparse AE showed superior disease outcome prediction. For clinical conclusions, scaling this approach to larger comparative studies with more diverse longitudinal data and simulation scenarios is needed to confirm the consistent superiority of this unified framework over other predictors.

### 5.2 P2 of ML and DL in AI

It introduced a novel dynamic fully Bayesian strategy to predict host status, case versus control, using publicly available longitudinal microbiome data from 16S marker gene surveys and metagenomics data with phylogenetic information.^[^ ^183^ ^]^ This predictor showed superior or comparable performance with faster computing time compared to those identified in their previous work^[^ ^198^ ^]^ and other conventional tools, including RF. This Bayesian neural network strategy in Python may enable accurate predictions of disease states/subtypes with multi‐class groups in longitudinal microbiome data, as well as case‐control designs. Future studies are expected to evaluate this method against other non‐Bayesian advanced ML and DL methods, including sparse AE and LSTM of P1.

### 5.3 P3 of ML and DL in AI

phyLoSTM is an enhanced dynamic DL‐based unified framework that integrates (1) an advanced feature selection scheme using a phylogenetic hierarchical tree and abundant patterns at the phylum level based on convolutional neural networks (CNNs) with (2) LSTM networks.^[^ ^164^ ^]^ To address imbalanced zigzag sample sizes between different groups before LSTM prediction, padding, and masking techniques were implemented. This method was evaluated using simulation and real data applications, including food allergies and preterm delivery modes for infants, demonstrating superior performance compared to RF and conventional regression‐based classifiers. However, rigorous assessment of the robustness of pre‐steps on the predictive power of final outcomes is still needed, based on uniformly standardized pre‐processing procedures for feature selection, missing value imputation, and sample size imbalance, utilizing various longitudinal microbiome data and multiple dynamic predictors.

### 5.4 P4 of ML and DL in AI

A recently developed advanced prediction framework for host status, based on dynamic longitudinal microbiome profiles, integrates several analytical pipeline steps.^[^ ^184^ ^]^ These include (1) imputing missing data for individual samples across multiple time points and normalizations, (2) feature engineering, (3) various feature selection strategies, and (4) host status prediction in a Python environment using phylogenetic trees, sample metadata, and temporal abundance profiles. Validation studies indicate its superior performance compared to conventional classifiers, though RF remains comparable. Effective data management during pre‐processing, to account for biological influences on prediction accuracy, is crucial for improving prediction rates and eliminating erroneous signals. However, currently, no universal consensus on generalized dynamic analytical predictor protocols with sophisticated pre‐steps has been reported.

### 5.5 P5 of ML and DL in AI

The contextualized DL predictor with transfer learning models, termed microDELTA, has been evaluated against widely used state‐of‐the‐art predictive models such as RF and neural network (NN) approaches.^[^ ^171^ ^]^ This study utilized publicly available longitudinal microbiome data, including gut microbiota from infants with different delivery modes and travelers experiencing long‐term dietary changes. The DL transfer modeling approach generally demonstrated superior predictive power, though this was heavily influenced by sample sizes, highlighting the need for extensive comparative studies with varied sample sizes and time points. This underscores the importance of balanced clinical/experimental designs and power tests to select appropriate time points and sample sizes, thereby minimizing biased predictions. Inconsistent results from the dynamic predictor due to varying parameters could pose reproducibility issues across studies.^[^ ^81^, ^182^ ^]^

### 5.6 P6 of ML/DL/AI

A unified dynamic framework combining CNN for feature selection and LSTM has been proposed, incorporating self‐distillation to identify interim and final phenotype predictions.^[^ ^71^ ^]^ Although it performs well, a thorough comparison is needed to determine which advanced dynamic DL methods (including dynamic P1 to P6) are most effective for analyzing longitudinal microbiome data in personalized and precision medicine for critical patient care and various disease progressive models.

***To sum up, with a brief of ML and DL predictors in*** ***section 4***, the results from P1 to P6 suggest that LSTM is generally more suitable for longitudinal microbiome time series data, while RF remains a comparably popular method. However, comparative studies and systematic reviews on dynamic ML and DL methods in AI are limited in scale and scope,^[^ ^49^, ^62^, ^132^, ^150^, ^153^, ^155^, ^190^, ^191^, ^199^, ^200^ ^]^ particularly concerning initial diagnosis, prognosis, progression, disease outcome prediction, patient monitoring, and therapeutic effects (see Tables S1 and S3, Supporting Information). Previous studies have not adequately addressed the biological effects of pre‐steps involved before and after microbiome quantification for accurate prediction outcomes.^[^ ^50^ ^]^ Collectively, these findings suggest that investigators should prioritize controlling non‐negligible noise sources throughout the workflow (see Figure 2), despite the availability of advanced denoising AI methods for health science predictions.^[^ ^62^, ^150^, ^164^, ^184^, ^189^, ^201^ ^]^ These discussions provide insights into the advanced dynamic ML and DL methods in supervised predictors. In the next sections (**Sections 4.7–4.10**), we discuss causal inferential network modules in ML tools, specifically dynamic Bayesian network (**DBN**) modules, designed for longitudinal temporal microbiome data.

### 5.7 DBN1 in ML

The gut microbiota of preterm infants was analyzed to infer **DBNs** among bacterial taxa, clinical variables like gestational and post‐conceptual ages, and community compositional transitions.^[^ ^185^ ^]^ Predictions included the impact of rare taxa on dominant bacteria and the correlation of abnormal patterns with dysbiosis in early infant microbiomes.^[^ ^185^ ^]^ However, the DBN method needs enhancement to incorporate intra‐/inter‐sample variability based on personalized medicine. It should also address multi‐omics levels with directed reciprocal effects rather than limiting to microbiome taxa‐based disease associations in temporal dynamics.^[^ ^182^, ^202^, ^203^ ^]^

### 5.8 DBN2 (PALM) in ML

Existing DBN methods infer modules based on temporally abundant microbial profiles, regardless of differing time‐varying trajectories influenced by external factors like antibiotic use. To address this, temporal aligner techniques^[^ ^144^, ^204^ ^]^ have been applied to longitudinal microbiome data, explicitly considering individual inter‐heterogeneity. The aligned temporal dynamics are then used to infer **DBN**s. This study proposes a novel DBN to infer directed acyclic graphs of network modules, accommodating heterogeneous multi‐omics longitudinal microbiome data for a single cohort.^[^ ^154^ ^]^ Improved temporal alignment incorporates gene expression information, unlike previous work limited to taxonomic data.^[^ ^144^ ^]^ The multi‐omics longitudinal human microbiome data includes five entities: taxa, microbial genes, metabolites, host gene expression, and environmental meta‐profiles. The learned directed network modules depict relationships across these entities over successive time stages by estimating temporal dynamic directional relationships through additional interim layers of gene and metabolite information (e.g., taxa (previous stage) → genes → metabolites → taxa (current stage)). In contrast, taxa‐specific DBNs only characterize taxa (previous stage)‐to‐taxa (current stage) relationships as partial conclusions. The known DBN modules with multi‐omics entities were validated using curated databases, while additional experimental work confirmed the impact of metabolites on significant taxonomic abundance growth over time for novel biomarkers, supporting biological causal relationships of the hypotheses. This emphasizes the necessity for larger, high‐quality databases to encompass both known and novel feature‐based signatures of taxa‐to‐genes and taxa‐to‐metabolites represented by identified DBN modules, as current databases provide limited information. Additionally, further experimental validation by biologists and clinicians is needed to ensure the confidence in estimating DBNs, given the lack of comprehensive prior knowledge compared to supervised dynamic ML and DL predictors. Therefore, this dynamic multi‐omics DBN analytical protocol for longitudinal microbiome data requires further evaluation with future‐generated multi‐omics longitudinal datasets. The biological impact of pre‐steps must be well‐qualified to establish inter‐dynamic study comparisons in this field's future. A subsequent study showed the results of the DBN analytical protocol with pre‐processing by comparing various feature selection tools on the same inflammatory bowel disease dataset used in DBN2, utilizing custom Python scripts.^[^ ^182^ ^]^

A recently developed dynamic ML dimensional reduction method, Essential Microbiome Dynamics (**EMBED**), was validated using real data and several synthetic datasets.^[^ ^205^ ^]^ This method is based on non‐linear tensor factorization, inferring ecological normal modes that share universal temporal dynamics of taxonomy across individuals during external perturbations, as well as unique subject‐specific patterns. It identifies a few independent orthogonal feature sets as major contributors to mainstream analyses, outperforming methods like compositional tensor factorization and singular vector decomposition. However, as recent feature selection methods like sparse AE and CNN have shown better predictor performance, this reduction technique for dynamic longitudinal microbiome data should be evaluated alongside these approaches.

### 5.9 Web Application TIME in ML

This study introduces a user‐friendly web server (**TIME**) featuring exploratory visual analyses, grouping similar temporal variation patterns indicative of dynamic perturbations, and interactive modules based on causality estimated by the longitudinal‐specific Granger causality method.^[^ ^206^ ^]^ Presently, this method is limited to a single series of time‐course microbiome data. Future enhancements should include more complex longitudinal microbiome data types with multiple classes and multi‐omics data.

### 5.10 MC‐TIMME in ML

The Bayesian non‐parametric generative probability method, Microbial Counts Trajectories Infinite Mixture Model (**MC‐TIMME**), implemented in MATLAB, characterizes temporal dynamics of microbial abundances.^[^ ^207^ ^]^ It estimates prototype groups representing temporal patterns using MCMC simulations and infers coordinated microbial signatures interacting based on host microbiology mechanisms, such as autoimmune responses to metabolic pathways after repeated perturbation. Converting this method to a R/Python package would enhance accessibility and compatibility with numerous R/Python packages, facilitating reproducible conclusions across different studies.^[^ ^53^ ^]^

***To sum up***, the inference of directed DBN modules as potential biomarkers is crucial for integrative strategies at multi‐omics levels in longitudinal microbiome data, enabling comprehensive pathological characterization.^[^ ^3^, ^182^ ^]^ Current leading‐edge dynamic approaches lack standardized analytical protocols for such data. Unified analytical protocols outperform marginal methods by simultaneously incorporating error‐prone factors across steps, in other words, separating pre‐steps and dynamic analyses.^[^ ^25^, ^149^, ^208^ ^]^ Available unified dynamic frameworks include (1) MMUPHin for longitudinal differential abundance tests and clustering with integrated pre‐processing for batch correction and zero inflation,^[^ ^44^ ^]^ (2) predictors (P1/P3/P4/P6) incorporating feature selection and missing data imputation,^[^ ^71^, ^164^, ^184^, ^195^ ^]^ and (3) DBN2 with an improved temporal aligner technique.^[^ ^154^ ^]^

**Summarizing advanced dynamic methods in** **sections** **3** **and** **4**, unlike unified dynamic analytical protocols, typical two‐step dynamic methods require separate pre‐step procedures. The choice of pre‐processing tools significantly affects the results of dynamic methods. Therefore, unified dynamic analytical protocols should be developed to minimize the biological impact of feature selection techniques, missing value treatments, uneven sample sizes, batch corrections, and zero inflation inferences on final outcomes.^[^ ^44^, ^50^, ^149^ ^]^ This will foster consensus across dynamic studies in the expanding field of microbiome‐based pathological medical research in personalized and precision medicine.^[^ ^51^, ^53^, ^150^ ^]^ Otherwise, superior performance without considering pre‐step effects is meaningless and merely superficial.

We recommend unified dynamic analytical protocols for various longitudinal microbiome data and metadata, along with authentic data‐driven validations as best practices. This approach examines the extent of pre‐step data management presented by subsequent results using multiple dynamic methods within analytical pipelines. Beginning with the selection of a widely cited reference baseline dynamic analytical protocol with proven or comparable performance could be defined as the most popular method with demonstrated asymptotic robustness from previous practices. Data‐driven validations of multiple step‐wise analytical protocols using dynamic methods and unified protocols chosen by researchers can be conducted, as exemplified in a large‐scale benchmarking study.^[^ ^177^ ^]^ However, this study is limited to static multi‐omics microbiome data and focuses primarily on differential abundance test methods.^[^ ^177^ ^]^ This concept can be extended to more complex longitudinal microbiome multi‐omics data and the dynamic univariate feature‐by‐feature longitudinal differential abundance test methods and feature‐to‐feature approaches as predictors and DBNs we discussed.

With the selected baseline reference method, choosing other candidates of some enhanced methods (at least three) presenting improved performances in the previous literature compared to the first selected most popular method could be included for the data‐driven validation procedures.

The findings from each protocol should be explored in terms of biological insights and statistical outcomes through interactive communication between biologists and analysts. For instance, compare generated lists of top candidate features across different longitudinal differential abundance test methods to assess similarity/dissimilarity and the biological impact of data management.^[^ ^50^, ^149^, ^177^ ^]^ Specifically, the combat **(Baseline) batch correction tool** can be used with Harman, percentile normalization and BDMMA.^[^ ^42^, ^43^, ^169^, ^179^ ^]^ Each batch tool should provide corrected abundance tables for subsequent analyses rather than merely identifying batch sources for exploratory data mining. MaAsLin2 and ancom2 **(Both baselines) longitudinal differential abundance test tools** can be used alongside other improved candidates and zero‐inflated modeling strategies.^[^ ^58^, ^143^, ^160^ ^]^ For predictive classifiers, RF and LSTM **(Both baselines) prediction models** with other dynamic predictors (temporal CNN and Bayesian P2), and **DBN2 (Baseline) versus BN methods** ^[^ ^71^, ^164^, ^183^, ^186^, ^195^ ^]^ can be used.

No optimal selection criteria exist for various dynamic methods, normalizations, batch corrections, feature selection tools, or missing value imputation tools for repeatedly measuring individual samples over time with microbial counts.^[^ ^62^, ^141^, ^144^, ^209^ ^]^ Normalized abundance data of microbiome profiles can still suffer from significant data contamination due to systematic artifacts from data aggregation. The appropriate normalization method and the validity of using log‐transformed data on post‐normalization for microbial compositional counts remain unclear.^[^ ^161^, ^178^, ^200^ ^]^

Data‐driven validation procedures are essential for dynamic studies, particularly when moderate efforts are applied. Unlike simulation‐based studies, longitudinal differential abundance tests without prior knowledge can be robustly validated by examining the top candidate features from leading dynamic analytical protocols. This involves assessing the biological impact of pre‐settings in clinical, experimental, and analytical protocols. Validation can be performed through multiple criteria: (1) concordant and discrepant metrics such as meta p‐values^[^ ^210^ ^]^ and intersected cardinality^[^ ^177^ ^]^; (2) goodness of fit tests by comparing estimated versus observed values from various dynamic methods, with or without zero‐inflation factors; (3) Type I error control rates by analyzing longitudinal differential abundance results, splitting the control group into sub‐groups through random shuffling after temporal alignments using mock samples to detect falsely temporally differentially abundant features^[^ ^177^ ^]^; and (4) functional enrichment analyses of top candidate features to confirm whether taxa with statistically significant temporal changes are biologically linked to functional pathway roles relevant to the phenotype of interest.

To assess prediction accuracy, given known sample labels, we can easily examine the biological impact of preliminary steps on final prediction results by comparing them to various advanced dynamic predictors chosen by investigators. Identified DBN modules across different analytical protocols can also be evaluated by mapping them to curated databases and conducting additional experimental work if applicable, as demonstrated in DBN2.^[^ ^154^ ^]^

When prior knowledge of true temporally differential abundances or DBN modules in multi‐omics longitudinal microbiome data is unavailable and standard dynamic analytical protocols are lacking, data‐driven validation procedures are crucial as best practices in analyzing longitudinal microbiome data. Additionally, well‐documented summary reports of data‐driven comparisons of various pre‐step tools and mainstream dynamic methods are valuable for ensuring reproducibility across dynamic microbial studies. We also anticipate the development of improved dynamic analytical protocols, supported by large‐scale validations and systematic comparative studies on quality controls at pre‐steps for clinical, experimental, and analytical protocols in future studies, akin to MBQC phase II.

To comprehensively address the lack of rigorous validation, future large‐scale systematic comparative studies should be conducted methodically. **First, *with the aspects of selection criteria***, researchers should consider multiple pre‐processing tools before employing dynamic methods, such as longitudinal differential abundance tests, predictive models, and DBNs, to assess their impact on analyses. **Second, *with aspects of various realistic scenarios***, these studies should include both real clinical data and synthetic datasets to ensure generalizability.^[^ ^25^, ^45^, ^48^, ^71^, ^72^, ^150^, ^153^, ^155^, ^183^, ^188^, ^190^, ^191^, ^192^, ^194^, ^195^, ^199^ ^]^ **Third, *from the perspective of wise practices*** of dynamic ML and DL methods in AI, this should balance the interpretability and prediction accuracy of disease outcomes. **Fourth, *from the perspective of system biology*,** temporal associations in causal inferences should be defined using multi‐omics dynamic longitudinal microbiome data.^[^ ^3^, ^211^ ^]^ **Last, *to build a reliable test model***, training and test samples should remain separate, exemplified by the use of the second period of a longitudinal cohort study as test data and the first baseline term as training data.^[^ ^191^ ^]^

Large‐scale validations will expedite longitudinal microbiome studies, aiding in the identification of pathogenesis, including carcinogenesis, across various temporal and spatial spectra of human diseases through advanced dynamic approaches in personalized and precision medicine. Additionally, it allows for efficient comparison of different studies through universal clinical, experimental, and analytical protocols for dynamic multi‐omics data.

## 6 Promise and Challenges of Longitudinal Multi‐Omics Microbiome Data Integration

**Figure 5:**
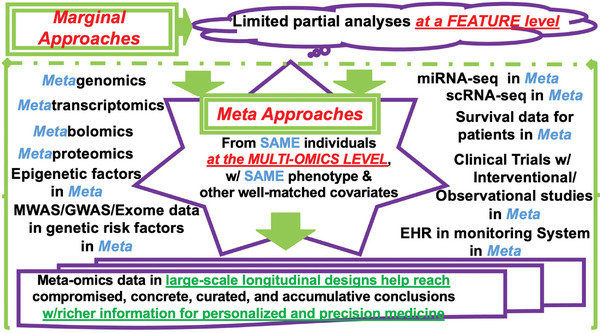
Multi‐star lightening layers in meta strategies in longitudinal time series cohort studies in personalized and precision medicine and therapeutic effects. Abbreviations: EHR (Electronic Health Record); GWAS (Genome‐wide Association Study); MiRNA‐seq (microRNA‐seq); MWAS (Microbiome‐wide Association Study); scRNA‐seq (Single Cell RNA‐seq).

**Figure 6:**
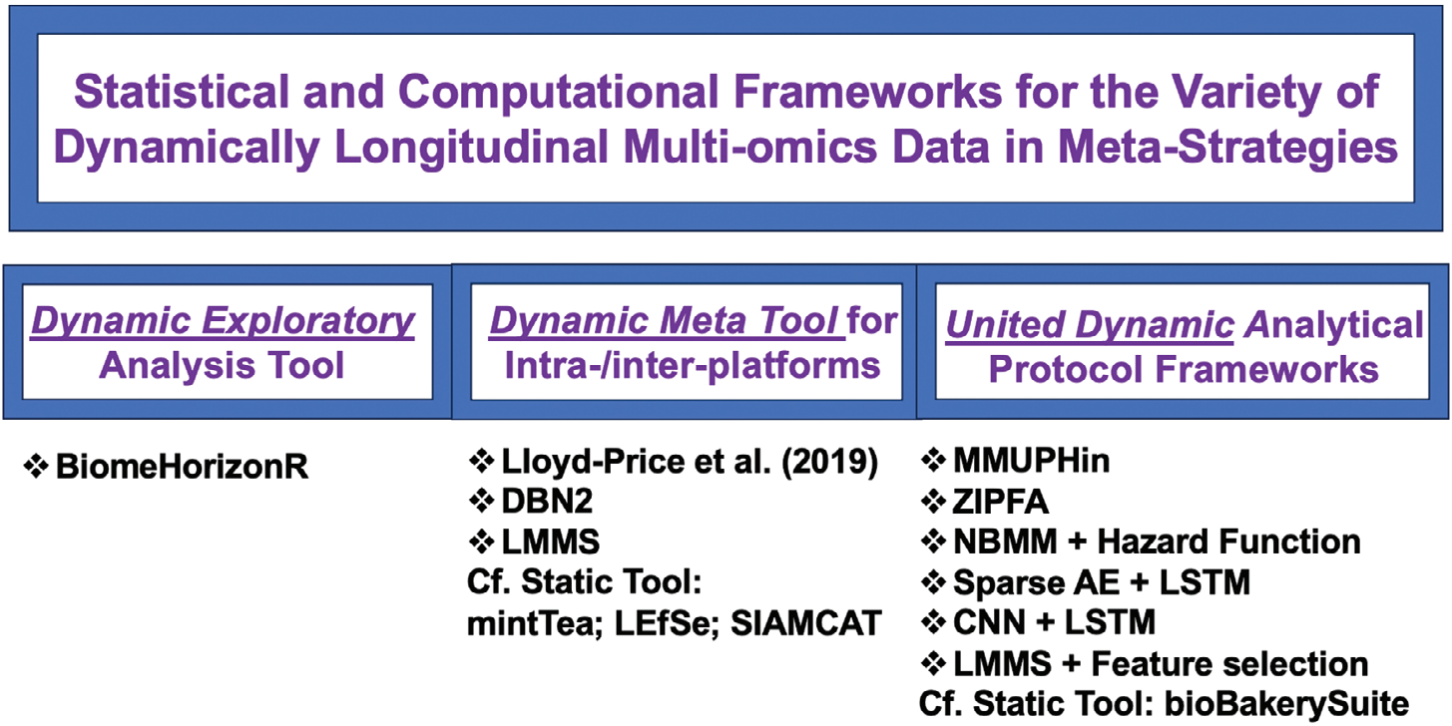
Unified dynamic methods in statistical and computational analytical protocols for longitudinal microbiome data and multi‐omics tools in meta‐strategies. Abbreviations: LEfSe (Linear Discriminant Analysis Effect Size); MMUPHin (Meta‐Analysis Methods with a Uniform Pipeline for Heterogeneity in microbiome studies); NBMM (Negative Binomial Mixed Effects Model); ZIPFA (Zero‐Inflated Poisson Factor Analysis).

With rapid advancements in biomedical technologies, it is now possible to characterize multi‐omics biomarkers using longitudinal studies in personalized and precision medicine.^[^ ^212^ ^]^ In such studies, it is promising to generate longitudinal multi‐omics data from the same subjects, including host gene expression, effects of interventions like drugs, diets, and antibiotics in metagenomics, metabolomics, microbiome‐epigenetic factors on diseases, metaproteomics, and scRNAseq data (**Figure** **5**). However, despite the fast pace of data generation, the development of dynamic methods for integrating multiple heterogeneous multi‐omics layers remains underdeveloped. Nonetheless, a few research groups have proposed dynamic methods targeting longitudinal multi‐omics microbiome data,^[^ ^41^, ^154^, ^182^, ^213^, ^214^ ^]^ moving beyond static meta strategies used for cross‐sectional multi‐omics microbiome data (**Figure** **6**).^[^ ^3^, ^177^, ^215^, ^216^ ^]^

### 6.1 Dynamic Meta Methods

#### 6.1.1 Lloyd‐Price et al

Developed a dynamic meta‐strategy utilizing WGS data from multiple body sites (stool and oral cavity) to define temporal dynamics at various levels: abundances, strains, and functions of microbial diversity through Gaussian process dynamics decomposition.^[^ ^41^ ^]^ The inferred components include noisy fluctuations, inter‐subject variability between body sites, and intra‐subject specific variations indicating time‐varying trajectories for each sample. However, this approach requires further integration with longitudinal microbiome compositional counts, addressing sparsity and batch contamination from multiple data sets. Beyond single‐layer WGS integration, dynamic meta‐methods for inter‐platform multi‐omics data integration are needed.^[^ ^154^, ^182^ ^]^

Variants of meta‐strategies have been frequently proposed in meta‐data, meta‐methods, meta‐protocols, meta‐studies, meta multi‐omics data, and meta‐assessment metrics such as meta p‐values, though their usage varies significantly. For instance, a study demonstrated vertical and horizontal integration of multi‐omics data including traditional arrays and sequencing based gene expression, methylation data, and proteomics data (excluding microbiome data), through the analytical workflow software in miodin, an R package.^[^ ^214^ ^]^ Vertical integration combines different molecular omics with multi‐omics platforms, while horizontal integration combines multiple datasets within a single‐omics platform.

**Figure 7:**
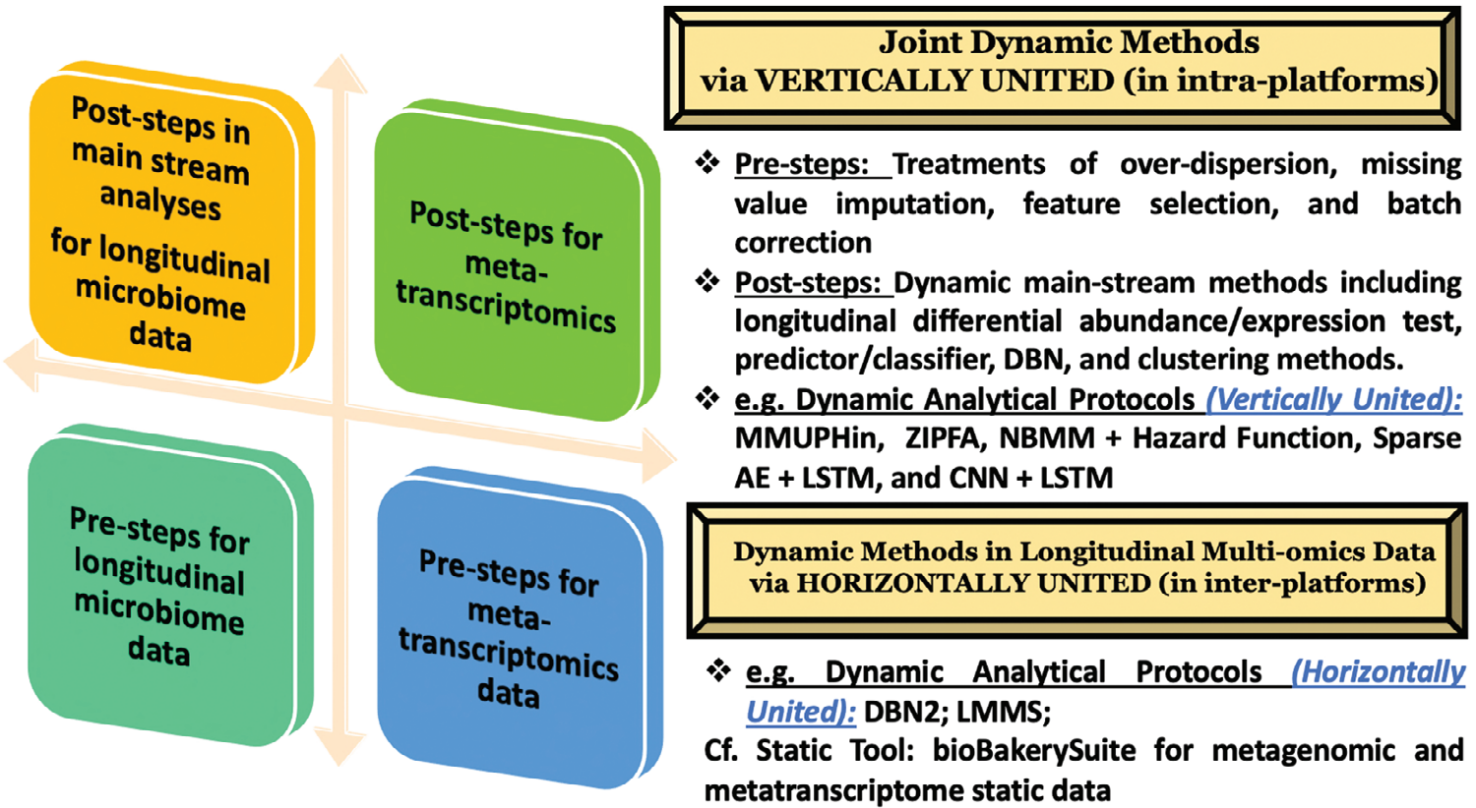
Meta dynamic methods in statistical and computational analytical protocols for longitudinal microbiome data and multi‐omics tools (e.g., bulk RNA‐seq).

In contrast, **Figure** **7** of our review represents the vertically integrated dynamic methods incorporated using multiple steps through a bottom‐up axis of the entire workflow within the longitudinal microbiome, bulk RNA‐seq, or other single‐layer datasets. However, horizontally integrated methods pertain to meta‐dynamic methods for longitudinal multi‐omics data in the axis of multi‐omics dynamic analytical protocols, which is exactly the opposite nuance in the description of miodin. Researchers must specify which components or datasets (samples, groups, main factors, and nuisance covariates) and protocols are integrated into their meta‐strategy.

Recently, a multi‐omics dynamic analytical framework based on linear mixed model splines (LMMS) and feature reduction techniques^[^ ^213^ ^]^ was developed to identify temporally changing features, accommodating uneven time points and sample sizes across entities. Validation through stringent assessment in comparative studies with real data applications and simulations is necessary for reliable performance in longitudinal microbiome studies in personalized and precision medicine.

Currently, DBN2 with temporal alignment methods and LMMS with feature reduction techniques for multi‐omics longitudinal data are the only dynamic meta‐protocols available for complex longitudinal designs.

We also included some static multi‐omics methods. For instance, the **bioBakery suite** integrates metagenomic and meta‐transcriptomic data,^[^ ^217^ ^]^ similar to the Tuxedo suite for bulk RNA‐seq data. It supports workflows from read quality controls to mainstream analyses, including microbial community identification, taxonomically abundant features, functional pathway metabolites, and strain‐specific analyses. However, this static suite needs enhancements for longitudinal cohort studies involving progressive disease models, treatments with varying effects, and other dynamic meta‐strategies.^[^ ^3^, ^132^, ^155^, ^218^ ^]^ **LEfSe,** another static multi‐omics tool, identifies microbial biomarkers across multi‐classes at abundance, expression, and functional levels using effect sizes and statistical significance, along with visualization tools.^[^ ^3^ ^]^

Similar to LEfSe,^[^ ^3^ ^]^ **mintTea** with intermediate integration has been implemented for identifying disease‐associated multi‐omics modules in static data comparing case versus control groups,^[^ ^215^ ^]^ excluding longitudinal datasets. It utilizes publicly available real WGS and metabolomics data. By analyzing normalized species abundance and log‐transformed metabolites, it identifies shared temporal modules across multi‐omics levels using sparse generalized canonical correlation transformations between two groups. It then finds groups representing similar module patterns through a resampling strategy. However, this method needs improvement to incorporate reciprocal directional relationships within modules across different meta‐features, rather than relying solely on correlations,^[^ ^154^, ^185^ ^]^ to better examine causative effects on highly enriched biomolecular functionalities in targeted diseases.

Given the rising interest in longitudinal multi‐omics data, advanced dynamic analytical protocols that accommodate multi‐modality at the omics level should be integrated into unified frameworks, ensuring flexibility, generalizability, and enhanced accuracy.

### 6.2 Longitudinal Multi‐Omics Data with Rich Information

Despite the potential of multi‐omics dynamic microbiome data, integrating such large datasets is challenging due to the complex network modules generated from heterogeneous data sets with varying experimental and analytical settings (Figure 7).^[^ ^154^, ^214^ ^]^ Recently, researchers have advanced microbiome‐disease association studies by incorporating spatial single‐cell resolution (scRNA‐seq) data, revealing different transcriptional rates across cells in heterogeneous microbial subpopulations, which bulk‐RNA‐seq data could not explore.^[^ ^40^, ^219^, ^220^, ^221^ ^]^ Moreover, studies using meta‐proteomics data and advanced technology have provided deeper insights into the functional roles of microbial communities by identifying metabolic enzymes and proteins.^[^ ^222^, ^223^, ^224^, ^225^, ^226^ ^]^ These innovations pave the way for longitudinal microbiome studies involving larger cohorts, more samples per subject, and multiple time points. Rigorous validation is essential for applying these findings to personalized and precision medicine.

Future research should aim to elucidate different causal relationships from a systems biology perspective, moving beyond mere associations. This will help unravel known and novel biological pathways related to disease progression, which are influenced by changes in composition, function, metabolites, strains, and multi‐omics entities within large‐scale dynamic meta‐strategies.^[^ ^24^, ^41^, ^48^, ^49^, ^154^, ^182^, ^189^, ^214^, ^215^ ^]^

## 7 Microbiome Data in Disease Temporal Spectra

Microbiome data, including gut flora, are crucial for identifying abnormal microbial diversity patterns linked to host physiology, drug treatments, antibiotic use, and other variables in human diseases and public health.^[^ ^21^, ^26^, ^29^, ^30^, ^81^ ^]^ Diet significantly influences the host ecosystem by regulating microbial interactions, host status, and external stimuli.^[^ ^5^, ^9^, ^16^, ^17^ ^]^ Recent studies have examined how long‐term and short‐term dietary interventions affect microbial diversity and their associations with diseases like inflammatory bowel disease and diabetes.^[^ ^2^, ^9^, ^16^, ^18^ ^]^ Additionally, studies on precision nutrition suggest that dietary guidance considering intra/inter heterogeneity of subjects is important for future studies in human diseases and public health.^[^ ^14^, ^15^, ^17^ ^]^ However, conclusions should be carefully drawn for target populations with appropriate sample sizes.

Furthermore, the causal or consequential effects of specific microbiomes on disease phenotypes remain largely unknown, and comprehensive paradigms for the implications of significant changes in dysregulated functionalities on dysbiosis are lacking.

When discerning common temporal patterns in longitudinal microbiome data, it remains unclear whether these dynamics stem from the internal shifts of the initial human gut microbiome or external forcing factors such as disease stages or treatment effects over time. Distinguishing the effects of these two factors on temporal changes is challenging, yet their decomposed effects may relate to disease causes and consequences, warranting ongoing debate akin to the “chicken or egg” dilemma.^[^ ^202^, ^211^ ^]^

Researchers consistently investigate microbial biomarkers in host ecosystem dysregulations, encompassing human diseases, disease stages, immunity, and defense systems through infections and pathogens.^[^ ^11^, ^19^, ^30^, ^35^, ^77^, ^78^, ^81^, ^92^, ^96^, ^98^, ^100^, ^151^, ^156^, ^227^, ^228^ ^]^ Key characteristics of personalized medicine and pharmaceuticals in extensive longitudinal microbiome cohort studies include immunologic diseases, cancers, neuropsychiatric disorders, and microbiome‐therapeutic approaches (see Table S1, Supporting Information).

## 8 Discussion

In this comprehensive review, we mainly focused on current challenges in microbiome studies, emphasizing advanced dynamic methods for characterizing temporal biological processes. We also suggested future directions based on data‐driven approaches using these dynamic analytical protocols. Dynamic association studies typically identify various microbial community compositions and functional activities by examining interactions with multiple microbes, the host ecosystem (e.g., diurnal/circadian rhythms), and other environmental/demographic factors at different levels: operational taxonomic units, strains, and multi‐omics entities, including metabolites.

To accurately characterize disease‐associated temporal dynamics and their functional roles, the effectiveness of dynamic methods for longitudinal microbiome and multi‐omics data should be validated throughout the entire analytical process rather than at individual steps. This approach is akin to playing golf, where each shot affects the final score, meaning interim data from preliminary steps can significantly influence the final results in both feature‐by‐feature and feature‐to‐feature analyses **AFTER** microbiome counts are quantified by features by samples. These tasks are typically performed by biostatisticians, bioinformaticians, and developers (post‐steps indicated by purple rectangles in Figure 2). Our review aimed to assist them in identifying current challenges in existing advanced dynamic approaches to minimize erroneous results. Additionally, we sought to facilitate the establishment of universal conclusions regarding clinical, experimental, and analytical protocols for future dynamic microbiome datasets, ensuring reproducible and reliable outcomes across different studies.

Dynamic analytical protocols should explicitly control the biological impact of clinical/experimental settings and pre‐processing tools on subsequent procedures. Data contamination significantly affects final results, not only in quantified microbiome count data, the main focus of this review but also in the initial pre‐processing tasks involving biologists and clinicians **BEFORE** microbiome counts are quantified (see blue rectangles in Figure 2). This includes (1) the impact of DNA sample extractions and primers selection,^[^ ^50^, ^229^ ^]^ which can introduce unwanted systematic artifacts; (2) the necessity of negative control samples to determine true signals and background noise,^[^ ^55^, ^230^ ^]^ particularly for low microbial biomass samples to verify the true compositional diversity of microbial communities; and (3) various laboratory technical issues, including known and unknown batch effects, that can affect the accuracy of results due to different batch sources in the workflow.^[^ ^230^ ^]^

Compared to other high‐throughput data like microarrays and bulk RNA‐seq, we lack validation procedures, despite the rapid application of human microbiome research in personalized and precision medicine for disease diagnosis, critical patient care monitoring, and drug development. Few research groups emphasize the importance of effective data management throughout the workflow.^[^ ^55^, ^229^, ^230^ ^]^ MBQC Phase I focused on the biological impact of initial steps on quantification.^[^ ^50^ ^]^

The power test step for justifying appropriate sample sizes is crucial for enhancing prediction accuracy, improving true discovery rates of univariate longitudinal different abundance tests, and increasing the accuracy of unsupervised clustering and dynamic network modules in causal relationships for specific research hypotheses.^[^ ^52^ ^]^ However, this step is often neglected in many microbiome studies. This oversight becomes a significant issue in large‐scale longitudinal microbiome study designs, where lurking confounders can obscure true biological signals, underscoring the importance of selecting suitable time points and subject samples. Additionally, sample size is more influential than sequencing depth coverage.^[^ ^52^ ^]^

For a **SINGLE** longitudinal cohort study design, deriving comprehensive conclusions on temporal dynamics requires multi‐omics data for identical samples in meta‐strategies.^[^ ^53^, ^159^, ^202^ ^]^ The temporal dynamics of bacterial communities at the taxa level are only part of the overall molecular genetic processes, which interact with gene expression, transcription factors, and protein expression in translation with bioactive metabolite components. To better characterize temporal abnormal patterns in disease microbiome studies, more repertoires of concordant/complementary temporal changing modules across different feature levels are needed in multi‐omics data to understand disease etiology from a systems biology perspective.^[^ ^40^, ^159^, ^219^, ^220^, ^222^, ^223^, ^224^, ^225^, ^226^ ^]^

Consequently, we emphasized the significant aspects of diverse longitudinal microbiome study designs and their integrative multi‐omics strategies for future research in personalized and precision medicine in this comprehensive review.

## 9 Closing Remarks

Numerous microbiome studies^[^ ^4^, ^12^, ^21^, ^31^, ^48^, ^49^, ^75^, ^79^, ^102^, ^151^, ^153^, ^155^, ^157^, ^188^, ^190^, ^191^, ^231^ ^]^ have established diagnostic measures for initial patient screening, stratified patients by disease severity, and predicted therapeutic responses to drugs and surgeries. However, the field is still evolving. Despite its recognition, challenges remain in developing reliable microbiome‐based diagnostic and therapeutic models that consider the heterogeneity of patients and the adaptive sensitivity of the human microbiome to external stimuli over time in personalized and precision medicine. The absence of validated dynamic methods for longitudinal microbiome multi‐omics datasets is also a bottleneck. Future microbiome studies must address these challenges.

**First**, patient samples should be categorized into different stages and sub‐types of a particular disease, as well as control groups (normal, cautionary control samples marked for prevention). Disease progression mapping should be well characterized in initial designs and sample collections in clinical and translational research, as sicker individuals are more prone to higher mortality, lower efficacy in microbiome‐based therapies, reduced survival rates, and increased infection risk. Large‐scale microbiome‐based interventional longitudinal studies with multi‐omics data^[^ ^20^, ^21^, ^23^, ^24^, ^81^, ^89^, ^106^, ^107^, ^186^ ^]^ should include well‐defined target populations considering comorbidities, age, sex, ethnicity, and other covariates such as diet, antibiotic use, drugs, exercise, and alcohol/smoking. **Second,** in the era of modern microbiome and genomic research coupled with AI methods, careful assessment of results is crucial to avoid overconfidence in predictive models for patient care and characterization of microbiome associations in diseases and therapies. Regulatory guidelines for clinical protocols in biotherapeutics, including pre‐ and probiotics, should be rigorously documented with criteria for safety, efficacy, and quality over both short and long terms.^[^ ^12^, ^157^, ^228^, ^232^, ^233^, ^234^, ^235^ ^]^ Microbiome association studies for diseases and therapeutic effects must include fungi, archaea, viruses, and bacteria for comprehensive microbiome characterization.^[^ ^151^ ^]^ **Third,** standardized clinical, experimental, and analytical protocols should be validated through large‐scale benchmarking studies using diverse methods, datasets, and parameter settings. Longitudinal microbiome studies in personalized and precision medicine should provide detailed workflows, including sample preparation, data availability, and dynamic algorithms with clear version labels for reproducibility. Efficient computational methods are necessary to handle large longitudinal microbiome multi‐omics data. **Fourth,** comprehensive dynamic statistical and computational frameworks in R/Python (including Web applications) should address uncertainties and errors across all workflow steps, rather than focusing on single‐step methods. **Fifth,** harmonized collaboration among the microbiome industry, pharmaceutical companies, academic researchers, and government regulators is essential to ensure safety, quality, efficacy, and reliability in longitudinal microbiome multi‐omics studies in personalized and precision medicine.^[^ ^12^, ^15^, ^48^, ^87^, ^212^, ^228^, ^234^, ^235^ ^]^

## Conflict of Interest

The authors declare no conflict of interest.

## Author Contributions

V‐K.S.O. conceived this comprehensive review, and V‐K.S.O. and R.W.L. wrote the manuscript. There are no prior publications or submissions with overlapping information, including studies or patents. This manuscript has not been, and will not be, submitted to any other journal while it is under consideration. All authors have approved the final manuscript as submitted and agreed to be accountable for all aspects of this work.

## Supporting information



Supporting Information
