# Supplementary material for: Wise Roles and Future Visionary Endeavors of Current Emperor: Advancing Dynamic Methods for Longitudinal Microbiome Meta‐Omics Data in Personalized and Precision Medicine
Source: Adv Sci (Weinh). 2024 Nov 13;11(47):2400458. doi: 10.1002/advs.202400458 (PMC11653615; doi:10.1002/advs.202400458)
Supplement: Supplementary file 1 — Supporting Information [file ADVS-11-2400458-s001.docx]

**Supplementary Table 1. Key representative longitudinal microbiome studies and their main features**

| **Phenotypes** | **Authors** | **Main features**  **in datasets** |
| --- | --- | --- |
| **IBD** | **1. Lloyd-Price [**[**1**](#_ENREF_1)**]**  **2. Halfvarson [**[**2**](#_ENREF_2)**]**  **3. Pascal [**[**3**](#_ENREF_3)**]**  **4. Shaw [**[**4**](#_ENREF_4)**]** | - IBDMDB resource (from Five top research centers) in characterization of IBD etiology in multi-modal meta-omics data: metagenomics, metatranscriptomics, metaproteomics, metabolomics, viromics, and FC based on 132 subjects. - Meta-data from multi-medical centers (three pediatric and two adult cohort studies during one-year IBD dynamic spectrum with global time points). - Stool, biopsy, and blood sample collection - Longitudinal cohort studies at multi-omics levels of host, microbial, environmental, and genetic factors with group levels of control non-IBD, UC, and CD |
|  |  | - The largest longitudinal microbiome data coupled with FC data in meta-strategy. - Subjects (n = 137) with five group levels, healthy control (n = 9), UC (n = 60), and CD (n = 49) in IBD, LC (lymphocytic colitis, n = 4) and CC (collagenous colitis, n = 15) in other chronic inflammatory gastrointestinal disease. - Four-month sample collections during two-year longitudinal cohort studies resulted in eight time points including zero. |
|  |  | - A longitudinal cohort study in the Spanish population using 16S rRNA marker gene survey microbiome data. - A meta-strategy in 178 subjects in the longitudinal IBD Spain cohort validated with other cohort studies of different geographical regions: Belgium, the UK, and Germany. - Stool sample collection at four different time points in one one-year study period. - Characterization of taxonomic and functional changes in disease dysbiosis amongst health control group versus main disease groups, UC and CD. |
|  |  | - Longitudinal cohort study comprising 19 pediatric subjects with treatment-oriented inflammatory bowel disease (IBD) and 10 healthy control subjects. - One-year longitudinal prospective cohort design with approximately six time points (six microbiome and FC samples in the treatment group versus five microbiome and 6.5 FC samples in the control group) at the time of diagnosis and during the study. - 16S rRNA marker gene survey data coupled with FC samples. |
| **Infant Gut** | **1. La Rosa [**[**5**](#_ENREF_5)**]**  **2. DIABIMMUNE [**[**6**](#_ENREF_6)**]** | - Longitudinal study of premature infants with postconceptional age at birth (33–36 weeks). - 16S rRNA pyrosequencing microbial community data. - 59 subjects with stool samples for characterization of developmental progressive models of early infant microbial communities. |
|  |  | - Longitudinal microbiome profile data from the DIABIMMUNE project in three geographical regions to characterize the interactions between the host ecosystem and gut microbiome in immunity. - https://pubs.broadinstitute.org/diabimmune |
| **Vaginal Microbiome** | **1. Gajer [**[**7**](#_ENREF_7)**]** | - Longitudinal study for characterization of changes in vaginal microbial bacteria communities using 32 non-pregnant and reproductive-age women. - Two samples per week for 16 weeks for self-collection of mid-vaginal swabs. - Use of 16S rRNA marker gene survey data. |
| **Response to cancer therapy** | **1. Yi [**[**8**](#_ENREF_8)**]** | - Subjects (n = 115; 84 patients with locally advanced colorectal cancer measured before and after neoadjuvant chemoradiotherapy (nCRT) versus 31 healthy subject samples without malignancies). - Longitudinal study in a single clinical center. - Monitoring management system of patients with rectal cancer for response effects to nCRT as dichotomous outcome (responding or not) using 16S rRNA marker gene survey data with clinical information - A training prediction data with another validation set. |
| **Normal aging processes in the gut** | **1. Wilmanski [**[**9**](#_ENREF_9)**]** | - The largest longitudinal gut microbiome study based on normal aging people and survival prediction of older people with four-year follow-up. - Approximately 9,000 individual subjects integrated from three different cohort studies with 18- to 101-year age in longevity: Arivale (+18 age from WA and CA state), MrOS (+65 age from six clinical centers in the USA), and AGP. - Stool sample collection with 16S rRNA marker gene survey data. |

Abbreviations:

AGP (American Gut Project); DIABIMMUNE (Diabetic and Auto-Immune illness prophylactic); CD (Crohn's Disease); FC (Fecal Calprotectin); IBD (Inflammatory Bowel Disease); IBD CC (IBD Collagenous Colitis); IBD LC (IBD lymphocytic colitis); IBDMD (Inflammatory Bowel Disease Multi-Omics Databases); MBX (Metabolomes); MGX (Metagenomes); MrOS (the Osteoporotic Fractures in Men); MTX (Metatranscriptomes); nCRT (neoadjuvant Chemoradiotherapy); UC (Ulcerative Colitis); VX (Viromes);

**Supplementary Table 2. Feature-by-feature univariate dynamic methods for longitudinal microbiome data in various phenotypes**

| **Dynamic type** | **Name** | **Main features** | **Considerations**  **for future research** |
| --- | --- | --- | --- |
| **ALDAT**  **in ZIM** | **1. ZIGMM [**[**10**](#_ENREF_10)**]** | - A mixture of zero-inflated Gaussian distribution - Log-transformed data + css normalization - Univariate fashion assuming all microbial taxa are independent - Competing tools: Metastats, Xipe, KW test in LEfSe, DESeq2, and edgeR - Microbiome 16S dynamic real data and synthetic studies | - Application to meta longitudinal microbiome data (for intra- and inter-omics data) - Incorporation of unwanted systematic biases, such as batches - Incorporation of data handling such as missing values and zero-inflation pre-steps - Incorporation of multivariate techniques, such as feature selection when selecting a model for each feature |
|  | **2. FZINBMM [**[**11**](#_ENREF_11)**]** | - Zero-inflated negative binomial mixed modeling with fast efficient computing time - Univariate fashion - Competing tools: ZIGMM, LMM, and NBZIM - Microbiome 16S rRNA and whole shotgun real dynamic data and synthetic studies | - Application to meta longitudinal microbiome data (for intra- and inter-omics data) - Incorporation of unwanted systematic biases, such as batches - Incorporation of data handling such as missing values and zero-inflation pre-steps - Incorporation of multivariate techniques, such as feature selection when selecting a model for each feature |
|  | **3. ZIPFA [**[**12**](#_ENREF_12)**]** | - Zero-Inflated Poisson Factor Model - Multivariate manner to account for coordinately dependent microbial features - Accounting for unwanted systematic biases such as batch sources - Normalized counts, no need to log-transform - Competing tools: log-PCA, PSVDOS, and GLM-based ordination method with zero-inflated quasi-Poisson latent factor model - Microbiome 16S rRNA dynamic real data and synthetic studies | - Application to dynamic meta-longitudinal microbiome data in comparative studies |
|  | **4. Comparative study with ZIM's [**[**13**](#_ENREF_13)**]** | - A comparative study to evaluate zero-inflated methods, naïve distributional count methods, and hurdle models | - Lager comparative study of rigorous assessment by including zero-inflated models including ZIGMM and FZINBMM versus other count-oriented methods without zero-inflation factor - Various sets of repeatedly measured longitudinal designs of microbiome data or its meta-dynamic data |
| **ALDAT in UAPM** | **1. MMUPHin [**[**14**](#_ENREF_14)**]** | - A cutting-edge statistical and computational framework as the unified dynamic analytical protocol - Integration of (1) preprocessing steps of batch corrections with zero-inflation factor, (2) longitudinal differential abundance test, (3) an unsupervised strategy finding population-specific patterns of microbial communities - Dynamical meta-microbiome data integrated with multiple longitudinal cohort studies - Uniformly standardized analytical processes and well-validation procedures - 16S microbiome longitudinal and cross-sectional datasets and simulation tasks - Competing tools: quantile-normalization, Combat, uncorrected data, and BDMMA for batch tools; DESeq2 and edgeR for longitudinal differential abundance test | - Lager comprehensively systematic evaluation by including newly developed zero-inflated models and more batch tools including Harman - Incorporation of multiple batches and unidentifiable batch sources |
|  | **2. NBMM + Hazard function [**[**15**](#_ENREF_15)**]** | - Unified analytical Bayesian framework in R package integrated between negative binomial mixed effects model incorporating other covariates and hazard function - Inference of significant associations of longitudinal time-varying relative microbial abundance profiles, metadata, and time-to-event indicating disease onset based on estimated posterior log-hazard ratio values and disease-free probability on sub-model - Comparison with previously jointly integrated modeling approach and cox-hazard proportional model based on both real data application and simulations - Evaluation metric: Area under the receiver operating characteristic (auROC) | - A systematic comparative study including multiple longitudinal real microbiome datasets, disease types, and disease courses - Flexible and generalized unified analytical framework in Bayesian approaches targeting dynamic multi-omics data, especially focusing on disease-progressive models and therapeutic effects in interventional studies - Incorporation with sophisticated pre-steps such as missing values, unbalanced samples between control versus case, zero-inflation in sparsity before estimation of disease initiation time-point |
| **BM in ALDAT** | **1. Harman [**[**16**](#_ENREF_16)**]** | - PCA-based batch correction - Transcriptomic real data applications - Providing batch-corrected profiles for subsequent analyses - Allows both a known or unidentifiable batch - Competing tool: Combat | - Microbiome real data applications and various mimic scenario sets, especially using dynamic longitudinal datasets |
|  | **2. Percentile-Normalization [**[**17**](#_ENREF_17)**]** | - Percentile normalized by healthy control samples - 16S rRNA microbiome real data applications, but static - No simulation studies in the evaluation - Providing batch-corrected profile - Competing tools: Combat, limma, and non-normalized relative abundances | - Application to dynamic longitudinal microbiome cohort studies - Incorporation of dynamic meta-microbiome or meta-omics data - Simulations for method validation - Shotgun Metagenomics real data applications |
|  | **3. BDMMA [**[**18**](#_ENREF_18)**]** | - Bayesian multinomial Dirichlet regression batch correction approach - Evaluation based on Shotgun metagenomics real data applications and mimic simulation works - Accounting for over-dispersion and dependent structures across microbial taxa on compositionality - A known batch - Competing tools: Combat | - 16S microbiome real data applications - Incorporation of dynamic longitudinal multi-omics microbiome cohort studies - Incorporation of multiple batch factors and unknown batch sources - Incorporation of batch-corrected profile of microbial counts |
| **Current challenges for development of advancing univariate dynamic methods** | | - Treatment of zero-inflation, batch effects, missing value imputations, feature selection for dimensional reduction techniques, imbalance of samples sizes between group levels, intra-/inter-heterogeneity of individual subject samples, and compositional microbiome counts. - Quality control for the biological impact of pre-steps onto the final outcomes at main-stream analyses - The lack of comprehensively systematic benchmarking comparative studies in large-scales for the rigorous assessment of validations of dynamic methods - The absence of gold-standards of dynamic analytical protocols suggested for the variety of longitudinal microbiome data and its meta multi-omics data in feature-by-feature approaches | |

Abbreviations^a^:

ALDAT (Advanced LDAT, Longitudinal Differential Abundance Test); BDMMA (Bayesian Dirichlet-Multinomial regression Meta-Analysis); BM (Batch Methods); GLM (Generalized Linear Model); FZINBMM (Fast Zero-Inflated Negative Binomial Mixed Model); LMM (Linear Mixed Model); LDAT (Longitudinal Differential Abundance Test); MMUPHin (Meta-Analysis Methods with a Uniform Pipeline for Heterogeneity in microbiome studies); UAPM (Unified Analytical Protocol Method); ZIGMM (Zero-Inflated Gaussian Mixture Model); ZIM (Zero-Inflated Model); ZIPFA (Zero-Inflated Poisson Factor Analysis); ^a^Those inside the purple oval are discussed in the present review.

**Supplementary Table 3. Feature-to-feature dynamic methods for longitudinal microbiome and its multi-omics data in various phenotypes**

| **Dynamic type** | **Name** | **Main features** | **Considerations for future research** |
| --- | --- | --- | --- |
| **ML and DL in AI predictors** | **1. LSTM [**[**19**](#_ENREF_19)**]** | - First deep learning (DL) study with LSTM with Sparse auto-encoder for feature selection - Prediction of food allergies in early life (0–3 years) in longitudinal gut microbiome data of DIABIMMUNE real data application - Competing tools: HMM, Multi-Layer Perception NN, SVM, RF, and LASSO - Evaluation metrics: mRMR (minimum redundancy, maximum relevance) in feature selection criteria, auROC, and Matthew’s correlation coefficient | - Large-scale validation based on various longitudinal microbiome datasets with longer series and denser sampling collection; inclusion of other DL tools that are designed for dynamic longitudinal microbiome datasets - Required simulation works in validation procedures |
|  | **2. MDTIRE**  **in BNN [**[**20**](#_ENREF_20)**]** | - Microbiome differentiable interpretable temporal rule engine - Improved version of MTIRE with efficient computing time - GUI framework for both longitudinal 16S and metagenomics microbiome data - Bayesian neural network-based DL tool - Major component layers: Phylogenetic tree, time-varying temporal change, detection, and rule layers - Longitudinal microbiome real and semi-synthetic datasets - Competing tools: MITRE, RF, and L1 regularized logistic regression - Evaluation metrics: F1 score | - Large-scale comparative study employing other non-Bayesian available DL methods, e.g. LSTM and various feature selection criteria for prediction accuracy; Evaluation of the biological impact of the choice of pre-steps onto the final prediction - More various longitudinal microbiome data application examples - Multi-omics longitudinal data |
|  | **3. phyLoSTM [**[**21**](#_ENREF_21)**]** | - A jointly integrated DL framework between 1) a feature selection scheme with a phylogenetic hierarchical tree and co-existing patterns of abundances across microbial features based on convolutional NN and 2) a prediction model of LSTM - Accounting for imbalanced sample sizes between control versus case group - Accounting for zig-zag sample collection in repeated measurements over time - Two longitudinal real data applications (DIABIMMUNE and DiGiuilo data) and simulation tasks - Competing tools: RF, SVM, LASSO, and naïve Bayesian classifiers - Evaluation metrics: auROC | - Large-scale comparative study employing Bayesian available DL methods, e.g. MDTIRE and various feature selection criteria for prediction accuracy; Evaluation of the biological impact of the choice of pre-steps onto the final prediction - More various longitudinal microbiome data application examples - Multi-omics longitudinal data |
|  | **4. FE_GRU [**[**22**](#_ENREF_22)**]** | - An integrated analytical protocol dealing with treatments of missing values and inconsistent numbers of time-points for individuals, normalization, feature engineering, feature selection with unsupervised and supervised strategies, and prediction in a Python setting - A large-scale comparative study by proposing a gated recurrent neural network modeling approach with dynamically temporally longitudinal microbiome data and semi-synthetic datasets - Competing tools: 1) For different feature selection criteria, RF, PCA, L1, auto-encoder, recurrence neural network feature elimination, and no feature extraction, and 2) For prediction accuracy, MTIRE, RF, SVM, LSTM, and GRU with and without feature selection - Evolution metric: auROC | - Sophisticated pre-processing procedures to increase prediction accuracy in issues: missing values, inconsistent samplings, unbalanced sample sizes in the group levels, and noise-free data - Handling integrated dynamic longitudinal microbiome data such as inter-studies and multi-centers |
|  | **5. microDELTA [**[**23**](#_ENREF_23)**]** | - microDELTA (micro-based deep life trajectory): built-in baseline neural network model for general dataset and EXPERT transfer learning neural network - Real data applications including the DIABIMMUNE study - No simulation tasks - Competing tools: MTIRE and traditional classifiers including RF - Evaluation metrics: auROC | - Sophisticated pre-processing procedures to increase prediction accuracy in issues: missing values, inconsistent samplings, unbalanced sample sizes in the group levels, and noise-free data - Handling integrated dynamic longitudinal microbiome data such as inter-studies and multi-centers |
|  | **6. Weighted LSTM [**[**24**](#_ENREF_25)**]** | - A single-center longitudinal study with clinical information on liver transplantation patient - A large-scale retrospective clinical longitudinal design with approximately 30 years of sequentially measured time points between control versus treatment groups - Weighted LSTM accounting for imbalanced sample sizes between group levels - Competing DL tools: LSTM without incorporation of imbalanced samples, Recurrent NN, Temporal convolutional networks - Competing ML tools: RF, SVM, and simple regression-based classifiers including logistic regression - Evaluation metrics: auROC | - A direct comparative study of improved prediction DL tools such as phyLoSTM and FE_GRU for the variety of longitudinal microbiome data or its metadata |
|  | **7. CNN + LSTM [**[**25**](#_ENREF_26)**]** | - Another dynamic DL tool designed for longitudinal microbiome data - Combinatorial implementation between feature selection by convolutional NN and LSTM for identification of temporal dynamic patterns - Treatment of missing values - Throughout transfer learning, self-distillation of interim and final prediction outcomes of host status - Two longitudinal microbiome real datasets: DIABIMMUNE and PROTECT study data - No simulation works - Competing tools: LSTM and RNN - Evaluation metrics: auROC and F1 score | - A large-scale comprehensively systematic evaluation by additionally employing advanced DL tools - Variety of longitudinal microbiome datasets, its metadata, and simulation tasks |
| **ML and DL**  **in AI**  **(Missing imputation)** | **1. DeepMicroGen [**[**26**](#_ENREF_27)**]** | - Missing imputation method of RNN with generative adversarial network (GAN) targeting dynamic longitudinal microbiome data - Real data applications: DIABIMMUNE data and BONUS-CF study data - Simulation tasks with varied parameter settings - Competing tools: existing imputation methods such as MICE, LOCF, simple replacements, and autoencoder + bidirectional GAN - Evaluation metrics: MAE | - A large-scale comprehensively systematic evaluation of advanced DL tools by using multiple reference longitudinal microbiome datasets - Exploration of the biological impact of selection of different pre-steps (missing value imputations) onto the final prediction outcomes instead of simple MAE criterion |
| **ML and DL**  **in AI predictors**  **(Review article)** | **1. Bhat [**[**27**](#_ENREF_28)**]** | - A comprehensive review article for AI and ML focused on liver diseases by categorizing previous studies in terms of prediction types, raw data sets in case and control, cross-validation, evaluation metrics, and used ML tools - ML tools for monitoring the system of patients, survival outcomes, and disease recurrences in initial diagnosis and prognosis in personalized medicine | - Application to dynamic longitudinal microbiome data in liver diseases, its metadata, and meta-omics data - A large-scale comparative study to rank various ML tools in such real data application and simulation works |
|  | **2. Zand [**[**28**](#_ENREF_29)**]** | - A large-scale longitudinally measured meta-cohort study within consecutive time points of two-year (1^st^ year: baseline and 2^nd^ year: validation) - Evaluated ML tools: LASSO, Ridge regression, SVM, RF, and NN - Two-year follow-up study monitoring patients with Inflammatory bowel diseases for adverse effects based on electronic health records - Prediction ML models for individualized stratified patient care - Evaluation metrics: sensitivity, specificity, auROC, and Brier score | - Application to dynamic longitudinal microbiome data in IBDs, its intra-platform metadata, and inter-platform multi-omics data - A large-scale comparative study to rank various ML tools in real data application and simulation works - More enhanced ML tools specifically designed for longitudinal microbiome data discussed in our review - Applications to rigorous and heuristic feature selection strategies |
|  | **3. Marcos-Zambrano [**[**29**](#_ENREF_30)**]** | - A comprehensive systematic review article for devoted COST Action ML4Microbiome activities - Multiple disease types including metabolic and cancer microbiome data, but mostly focused on static datasets - Various ML tools, mainly categorized into supervised, unsupervised, and directed causal–consequent relationships - Discussed supervised ML tools: conventional methods including logistic regression, LDA, kNN, naïve Bayes classifier, SVM, and tree-based decision makers including RF - DL methods including CNN - Unsupervised clustering methods including Hclust, Biclustering, and non-negative matrix factorization - Directed causal–consequent relationships including Dynamical Bayesian Networks (DBNs) for longitudinal microbiome data with various external factors, Mendelian randomization, Correlation-based network analysis - ML applications focused on personalized monitoring systems of patient care in terms of initial diagnosis, prognosis, and therapeutic effects | - More enhanced ML tools specifically designed for longitudinal microbiome data discussed in our review - Applications to rigorous and heuristic feature selection strategies - Generalization of analytical protocols in preprocessing steps consequently affecting the outcomes of various ML tools |
|  | **4. Curry [**[**30**](#_ENREF_31)**]** | - A systematic review article for human gut microbiome data related to multiple disease types, gut–liver axis, IBD, obesity, and T2D - Mostly focused on static microbiome data - Reviewed ML frameworks in the following: ***A. MetAML framework*** targeting shotgun metagenomics data, incorporated with SVM, RF, LASSO, Elastic net, and incorporated with evolution metrics, overall accuracy, precision, F1, and auROC. ***B. PopPhy-CNN*** framework required for relative abundance at genus and species level, phylogenetic hierarchical tree information, and built-in house feature selection. ***C.*** ***Met2lmg framework*** incorporated with CNN, t-SNE feature selection, and it outperformed MetAML RF. ***D. MicroPheno framework*** for 16S marker gene survey data, incorporated with embedded k-mers prediction ML tool of MLP NN. ***E. MetaPheno framework*** incorporation with the extraction of k-mers and prediction ML tools, RF, SVM, XGBoost, gcForest, and AE-Deep NN. ***F. DeepMicro framework,*** evaluated with various AE strategies and prediction models for auROC including SAE/DAE/VAE/CAE plus RF, SVM, and MLP NN. ***G. MVIB (multi-modal variation bottleneck) framework of a deep NN*** to handle complex input data architecture containing multi-modality shown in outperformance to DeepMicro plus VAE | - Application to dynamic longitudinal microbiome data in IBDs, its intra-platform metadata, and inter-platform multi-omics data - A large-scale comparative study to rank various ML tools in real data application and simulation works - More enhanced ML tools specifically designed for longitudinal microbiome data discussed in our review - Applications to rigorous and heuristic feature selection strategies - Generalization of analytical protocols in preprocessing steps consequently affecting the outcomes of various ML tools |
|  | **5. Stafford [**[**31**](#_ENREF_32)**]** | - A systematic survey of best practices with AI tools for inflammatory bowel diseases, especially focused on each single level of study in microbiome, metabolomics, transcriptome, genetics, clinical data, medical imaging, etc. - Commentary Review for Big fast-moving trends in microbiome-based studies in personalized and precision medicine by presenting a numerously accelerating rate of increasing uses of NN tools in the last few years | - Best practical exemplary studies of AI tools with success and validation for temporally longitudinal microbiome data - Large-scale benchmarking studies at multi-omics levels: microbial-relevant and coordinately working biomarker signatures with metabolites, gene functions, and other molecular layers |
| **ML DBN** | **1. DBN.1 [**[**32**](#_ENREF_33)**]** | - Accounting for current state versus previous time-point based on Markov Chain - Use of publicly available large-scale longitudinal microbiome data of premature infant gut flora **(La Rosa study, See Table 1**) - Use of CGBayesNet R package - Inferences (1) time-varying changes in microbial compositions and (2) relationships between taxa and clinically important covariates for environmental factors including antibiotic uses, delivery modes, breastfeeding, day-of-life when samples are collected, and post-conceptual age - Accuracy metric: MAE | - Validation works based on multiple longitudinal real datasets and simulations - Comparisons with other ML dynamic network inference tools - Biological definitions of inferred DBNs by connecting to truly relevant interplays of biologically enriched/depleted pathways - Dealing with microbiome-specific compositional counts including zero-inflation and missing values before the prediction of DBNs - Applications of DBNs to dynamic multi-omics data |
|  | **2. DBN.2 [**[**33**](#_ENREF_34)**]** | - Before the inference of dynamic network modules, prerequisite preprocessing procedures are incorporated with the proposed DBN model including (1) filtering out taxa with zero and low abundances and (2) temporal alignment accounting for differently changing rates over time across individual subject samples by time-warping method - Inference of dynamic network relationships by DBN in the Markov Chain from two consecutive time-points - Use of an improved version of CGBayesNets R package accounting for intra-edges - Use of multiple publicly available longitudinal microbiome data in different body sites, infant gut, vaginal cavity, and oral cavity | - Instead of filtering out taxa with zero or low abundances, enhanced quantification tools with zero-inflated models - Development of better quantification considering longitudinal compositional microbiome counts - Handling pre-step analyses (missing value imputation and feature selection) before prediction of DBN - Large-scale comparative study with varied simulation scenarios - Biological definitions of inferred DBNs by connecting to truly relevant interplays of biologically enriched/depleted pathways - Applications to DBNs to dynamic multi-omics data |
| **ML TA**  **in DTW** | **1. ML temporal alignment [**[**34**](#_ENREF_35)**]** | - Comparison of dynamic time-warping temporal aligners in different scenarios by randomly shuffling individual subject samples with different time-varying trajectory rates - Use of four publicly available longitudinal microbiome datasets - Assessment metrics: Pearson correlation between predicted versus actual age | - Evaluation of subsequent DBN or other network inference tools on how such temporal alignments of dynamic time-warping tools affect subsequent results - Large-scale comprehensively systematic comparative study based on standardized analytical protocols for improved quantification, preprocessing procedures, temporal alignments, longitudinal different abundance tests, and dynamic network inferences |
| **ML FS**  **in DRI** | **1. ML Dimensional reduction technique [**[**35**](#_ENREF_36)**]** | - Dimensional reduction technique based on Non-linear tensor factorization - Inference of most highly contributing loading factors based on microbial taxa across time-varying individual subject samples - Inference of ecological normal modes defining universal and differentiated temporal dynamics across subjects - Validation with other existing tools: compositional tensor factorization and singular value decomposition | - Investigation of the impact of the choice of different feature selection methods on the final results of mainstream dynamic players: predictors and DBNs - Further investigation of the biological impact of other issues at pre-steps, e.g. data contamination due to batches and missing values onto the final results of mainstream dynamic players: predictors and DBNs - Application to multi-omics longitudinal data |
| **Web server application** | **1. ML TIME [**[**36**](#_ENREF_37)**]** | - Web-server application - Incorporation with dynamic time-warping and Granger causality for   (1) identification of temporally differentially changing taxa, (2) clustering of co-abundances in temporal dynamics, and (3) inference of Ganger causality based on abundance profile and metadata in a visualization manner | - Validation of identified modules representing temporal changes and causal inference tools in large-scale comparative studies compared to others - User-friendly Web Server Application, further incorporated with enhanced ML predictors and dynamic network inferences - Allowance of multi-omics longitudinal microbiome data |
| **ML Unsupervised clustering tool** | **1. ML MC-TIMME [**[**37**](#_ENREF_38)**]** | - A non-parametric hierarchical mixture Bayesian framework inferring signature diversity including prototype time-varying changing patterns across taxa, coordinately changing patterns in temporal dynamics, and estimation of equilibrium time-points after perturbated external factors such as antibiotics - Use of a publicly available microbiome dataset | - Conversion into R/Python package for compatibility with other ML tools - Evaluation with large-scale comparative study including multiple real and synthetic datasets by including different ML methods in the characterization of temporal dynamics - Incorporation with dynamic Bayesian network modules and biological insights at functional pathway levels - Incorporation with ML predictors for temporal dynamic microbiome data - Allowance of multi-omics longitudinal time course microbiome data |
| **ML DBN**  **(Comparative study)** | **1. Kodikara [**[**38**](#_ENREF_39)**]** | - Large-scale comparative study for ZIM and DBN models - A systematic evaluation of existing methods in longitudinal differential abundance test, unsupervised clustering, DBNs, and LVs in ML tools - Comparisons of relative and absolute abundances - Real data application of gut flora study during VREfm colonization and simulations - Zero-inflated models versus others - Conventional clustering techniques, including PCA and Hclust - Evaluation metrics: Sensitivity and specificity in LDAT and clustering accuracy | - Establishment of generalized golden-standard pre-processing and main-stream analysis analytical protocol incorporated with sophisticated normalization, missing value imputation, zero-inflation, batch correction, and imbalance of group sizes - Inclusion of various longitudinal microbiome datasets and dynamic multi-omics data - Large-scale comparative study with varied parameter settings |
| **ML DBN**  **(Review article)** | **1. Laccourreye [**[**39**](#_ENREF_40)**]** | - Large-scale comparative study of various BN and DBN ML tools by focusing on publicly available longitudinal and multi-omics microbiome data, incorporated with various feature selections in ad-hoc phyton scripts | - Inspection of the biological impact of pre-steps (feature selections, missing value imputation with zero-inflation) onto the inferences of DBNs |
| **ML and DL in AI for DMM**  **(CS & RA)** | **1. Seyed Tabib [**[**40**](#_ENREF_41)**]** | - A comprehensive survey of applications of various ML tools to clinical patient care with inflammatory bowel diseases - Applications of ML tools to screen patients during initial diagnosis and prognosis, including follow-up management of individualized response to treatments and recurrences, stratification of subtypes of diseases in severity - Survey of meta-omics data with multi-layers of different entities to better characterize the complex etiological underlying mechanisms in pathophysiology revealing up/downstream regulators as whole biomarker signatures in meta-strategies in the following - Transcriptional binding sites, epigenetic factors, genetic risk factors, protein-protein interactions, post-transcriptional events, miRNAs targeting gene expressions, metabolites, and other components by focusing on human gut microbiome data in inflammatory bowel diseases - Urgent need for available longitudinal microbiome data at the level of the public domain | - Establishment of various longitudinal microbiome data, its metadata across multiple studies, and its meta-omics data on biobank - Dynamical-specific DL tools designed for longitudinal microbiome data or its meta-omics data, reviewed in our current study - Validation of prediction models in DL tools, the evaluation of feature selection criteria, and choice of different selecting strategies - Examination of the impact of sophisticated pre-processing analytical tools: (1)a choice of different missing value imputation ML tools, (2) normalization methods and batch detection tools, and (3) various feature selection criteria - More available cell-specific studies in lineages (scRNAseq) other than whole bulk RNA-seq data - More available examples of metaproteomics data coupled with longitudinal microbiome data |
|  | **2. David [**[**41**](#_ENREF_42)**]** | - An overview of the central roles of DL transfer learning tools for integrated longitudinal microbiome data - Using DL tools, detection of inter-study specific golden and standardized biomarker signatures with generalization across multiple different studies - Accounting for inter-study variation, and noise-free input data in the data aggregation processes | - In addition to inter-study variation, DL tools consider intra-/inter-individual variability of heterogeneity in personalized and precision medical care - Dynamical-specific DL tools for longitudinal microbiome data or time course microbiome multi-omics data - Rigorous validation procedures of DL tools during initial diagnosis, prognosis, and therapeutic effects |
|  | **3. Li [**[**42**](#_ENREF_43)**]** | - Meta-strategies of various ML tools based on highly heterogeneous data with more complexity due to integration with different biological processes for human gut flora microbiome data - How to combine multiple rich data resources, (1) all-to-one low-level meta-strategy to generate a concatenate feature matrix for prediction model at once, (2) corresponding transformed feature set from each omics data to build a prediction model in parallelized intermediate level of meta-strategy, and (3) each level omics data builds its prediction model for final prediction in a high level of meta-strategy | - Demonstration of superior performances of DL tools in a wide range of meta-omics real datasets - A better solution for the lack of available dynamically processed longitudinal microbiome data or other types of dynamic meta-omics data - Dynamical-specific DL tools implemented suitably for multi-omics longitudinal microbiome data - Rigorous assessment of the robustness of various DL tools during initial diagnosis, prognosis, and therapeutic effects, especially when applying for data aggregation by carefully controlling the quality of pre-steps in the entire workflow |
|  | **4. Medina [**[**43**](#_ENREF_44)**]** | - An overview of various ML tools of applications to microbiome-specific research in the following:   (1) differently collected data types in experimental designs, static, spatial, and temporal processes (2) technical strategies of ML tools for pre-processing procedures with treatments for noise-free sets, detection of most highly contributing features by ML tools in dimensional reduction techniques, characterization of coordinately working features by unsupervised ML tools, and supervised prediction models of disease phenotypes   - Reviewed ML tools for prediction in microbiome data: RF, SVM, traditional fully connected NN (FCNN), and advanced NNs including CNN for spatial data, e.g. PopPhy-CNN framework, RNN for time course microbiome data, especially longitudinal data, e.g. pyLoSTM framework - Dimensional reduction DL tools: PCA, PCoA, tSNE, UMAP | - Inclusion of various ML tools for causal–consequent relationships at multi-omics data i.e. DBNs - Expansion of biobanks with available longitudinal data and its meta-omics data collected from a wide range of disease types, disease spectral courses, and individualized patient monitoring care systems with therapeutic short/long-term effects - Optimizing prediction ML tools in a larger-scale comparative study to evaluate every step in the following:   impact of (1) normalization and batch correction, (2) feature selection schemes, (3) cross-validations, and (4) choice of different ML including DL tools |

Abbreviations:

AE (AutoEncoder); AI (Artificial Intelligence); auROC (Area Under Receiver Operating Characteristic curve); BN (Bayesian Network); BNN (Bayesian Neural Network); BONUS-CF (Baby Observational and Nutrition Study-Cystic Fibrosis); CAE (Convolutional AE); CNN (Convolutional NN); DBN (Dynamical Bayesian Network); DIABIMMUNE (Diabetic and Auto-Immune illness prophylactic); DAE (Deep AE); DL (Deep Learning); DMM (Dynamical Meta-Omics data); DRT (Dimensional Reduction Technique); DTW (Dynamic Time Warping); GUI (Graphical User Interface); FE_GRU (Feature Extraction Gated Recurrent Unit); FCNN (Fully Connected NN); FS (Feature Selection); IBD (Inflammatory Bowel Disease); GAN (Generative Adversarial Network); HMM (Hidden Markov Model); gcForest (multi-Grained Cascade Forest); KW(Kruskal Wallis test); LASSO (Least Absolute Shrinkage and Selection Operator); LDA (Linear Discriminant Analysis); LOCF (Last Observation Carried Forward); log-PCA (log-Principle Component Analysis); LSTM (Long Short Term Memory); LV (Lotka-Volterra); MAE (Mean of Absolute Error); MDTIRE (Microbiome Differentiable Interpretable Temporal Rule Engine); metAML (METagenomic prediction Analysis based on MAchine Learning); MICE (Multiple Imputation by Chained Equations); microDELTA (microbial-based DEep Life Trajectory); MITRE (Microbiome Interpretable Temporal Rule Engine); ML (Machine Learning); MLP (Machine Learning Predictor); MVIB (Multi-modal Variation Information Bottleneck); NN (Neural Network); PSVDOS (Poisson Singular Value Decomposition with OffSet); RF (Random Forest); RNN (Recurrent NN); SAE (Shallow AE); SVM (Support Vector Machine); t-SNE(t-distributional Stochastic Neighbor Embedding); T2D (Type II Diabetes); UMAP (Uniform Manifold Approximation and Projection); VAE (Variational AE); VREfm (Vancomycin-resistant Enterococcus faecium);XGBoost (Extreme Boosting Decision Tree);

**References**

[1] J. Lloyd-Price, C. Arze, A. N. Ananthakrishnan, M. Schirmer, J. Avila-Pacheco, T. W. Poon, E. Andrews, N. J. Ajami, K. S. Bonham, C. J. Brislawn, D. Casero, H. Courtney, A. Gonzalez, T. G. Graeber, A. B. Hall, K. Lake, C. J. Landers, H. Mallick, D. R. Plichta, M. Prasad, G. Rahnavard, J. Sauk, D. Shungin, Y. Vázquez-Baeza, R. A. White, J. Braun, L. A. Denson, J. K. Jansson, R. Knight, S. Kugathasan, D. P. B. McGovern, J. F. Petrosino, T. S. Stappenbeck, H. S. Winter, C. B. Clish, E. A. Franzosa, H. Vlamakis, R. J. Xavier, C. Huttenhower, I. Investigators, *Nature* **2019**, *569* (7758), 655, <https://doi.org/10.1038/s41586-019-1237-9>.

[2] J. Halfvarson, C. J. Brislawn, R. Lamendella, Y. Vázquez-Baeza, W. A. Walters, L. M. Bramer, M. D'Amato, F. Bonfiglio, D. McDonald, A. Gonzalez, E. E. McClure, M. F. Dunklebarger, R. Knight, J. K. Jansson, *Nat Microbiol* **2017**, *2*, 17004, <https://doi.org/10.1038/nmicrobiol.2017.4>.

[3] V. Pascal, M. Pozuelo, N. Borruel, F. Casellas, D. Campos, A. Santiago, X. Martinez, E. Varela, G. Sarrabayrouse, K. Machiels, S. Vermeire, H. Sokol, F. Guarner, C. Manichanh, *Gut* **2017**, *66* (5), 813, <https://doi.org/10.1136/gutjnl-2016-313235>.

[4] K. A. Shaw, M. Bertha, T. Hofmekler, P. Chopra, T. Vatanen, A. Srivatsa, J. Prince, A. Kumar, C. Sauer, M. E. Zwick, G. A. Satten, A. D. Kostic, J. G. Mulle, R. J. Xavier, S. Kugathasan, *Genome Med* **2016**, *8* (1), 75, <https://doi.org/10.1186/s13073-016-0331-y>.

[5] P. S. La Rosa, B. B. Warner, Y. Zhou, G. M. Weinstock, E. Sodergren, C. M. Hall-Moore, H. J. Stevens, W. E. Bennett, N. Shaikh, L. A. Linneman, J. A. Hoffmann, A. Hamvas, E. Deych, B. A. Shands, W. D. Shannon, P. I. Tarr, *Proc Natl Acad Sci U S A* **2014**, *111* (34), 12522, <https://doi.org/10.1073/pnas.1409497111>.

[6] A. D. Kostic, D. Gevers, H. Siljander, T. Vatanen, T. Hyötyläinen, A. M. Hämäläinen, A. Peet, V. Tillmann, P. Pöhö, I. Mattila, H. Lähdesmäki, E. A. Franzosa, O. Vaarala, M. de Goffau, H. Harmsen, J. Ilonen, S. M. Virtanen, C. B. Clish, M. Orešič, C. Huttenhower, M. Knip, R. J. Xavier, D. S. Group, *Cell Host Microbe* **2015**, *17* (2), 260, <https://doi.org/10.1016/j.chom.2015.01.001>.

[7] P. Gajer, R. M. Brotman, G. Bai, J. Sakamoto, U. M. Schütte, X. Zhong, S. S. Koenig, L. Fu, Z. S. Ma, X. Zhou, Z. Abdo, L. J. Forney, J. Ravel, *Sci Transl Med* **2012**, *4* (132), 132ra52, <https://doi.org/10.1126/scitranslmed.3003605>.

[8] Y. Yi, L. Shen, W. Shi, F. Xia, H. Zhang, Y. Wang, J. Zhang, X. Sun, Z. Zhang, W. Zou, W. Yang, L. Zhang, J. Zhu, A. Goel, Y. Ma, *Clin Cancer Res* **2021**, *27* (5), 1329, <https://doi.org/10.1158/1078-0432.CCR-20-3445>.

[9] T. Wilmanski, C. Diener, N. Rappaport, S. Patwardhan, J. Wiedrick, J. Lapidus, J. C. Earls, A. Zimmer, G. Glusman, M. Robinson, J. T. Yurkovich, D. M. Kado, J. A. Cauley, J. Zmuda, N. E. Lane, A. T. Magis, J. C. Lovejoy, L. Hood, S. M. Gibbons, E. S. Orwoll, N. D. Price, *Nat Metab* **2021**, *3* (2), 274, <https://doi.org/10.1038/s42255-021-00348-0>.

[10] J. Paulson, H. Talukder, H. Bravo, Longitudinal differential abundance analysis of microbial marker-gene surveys using smoothing splines. Cold Spring Harbor Laboratory: bioRxiv, **2017**.

[11] X. Zhang, N. Yi, *Bioinformatics* **2020**, *36* (8), 2345, <https://doi.org/10.1093/bioinformatics/btz973>.

[12] T. Xu, R. T. Demmer, G. Li, *Biometrics* **2021**, *77* (1), 91, <https://doi.org/10.1111/biom.13272>.

[13] L. Xu, A. D. Paterson, W. Turpin, W. Xu, *PLoS One* **2015**, *10* (7), e0129606, <https://doi.org/10.1371/journal.pone.0129606>.

[14] S. Ma, D. Shungin, H. Mallick, M. Schirmer, L. H. Nguyen, R. Kolde, E. Franzosa, H. Vlamakis, R. Xavier, C. Huttenhower, *Genome Biol* **2022**, *23* (1), 208, <https://doi.org/10.1186/s13059-022-02753-4>.

[15] P. N. Luna, J. M. Mansbach, C. A. Shaw, *PLoS Comput Biol* **2020**, *16* (12), e1008473, <https://doi.org/10.1371/journal.pcbi.1008473>.

[16] Y. Oytam, F. Sobhanmanesh, K. Duesing, J. C. Bowden, M. Osmond-McLeod, J. Ross, *BMC Bioinformatics* **2016**, *17* (1), 332, <https://doi.org/10.1186/s12859-016-1212-5>.

[17] S. M. Gibbons, C. Duvallet, E. J. Alm, *PLoS Comput Biol* **2018**, *14* (4), e1006102, <https://doi.org/10.1371/journal.pcbi.1006102>.

[18] Z. Dai, S. H. Wong, J. Yu, Y. Wei, *Bioinformatics* **2019**, *35* (5), 807, <https://doi.org/10.1093/bioinformatics/bty729>.

[19] A. A. Metwally, P. S. Yu, D. Reiman, Y. Dai, P. W. Finn, D. L. Perkins, *PLoS Comput Biol* **2019**, *15* (2), e1006693, <https://doi.org/10.1371/journal.pcbi.1006693>.

[20] V. S. Maringanti, V. Bucci, G. K. Gerber, *mSystems* **2022**, *7* (5), e0013222, <https://doi.org/10.1128/msystems.00132-22>.

[21] D. Sharma, W. Xu, *Bioinformatics* **2021**, *37* (21), 3707, <https://doi.org/10.1093/bioinformatics/btab482>.

[22] X. Chen, L. Liu, W. Zhang, J. Yang, K. C. Wong, *Brief Bioinform* **2021**, *22* (6), <https://doi.org/10.1093/bib/bbab223>.

[23] a) H. Zhang, H. Chong, Q. Yu, Y. Zha, M. Cheng, K. Ning, *Brief Bioinform* **2023**, *24* (1), <https://doi.org/10.1093/bib/bbac629>; b) H. Chong, Y. Zha, Q. Yu, M. Cheng, G. Xiong, N. Wang, X. Huang, S. Huang, C. Sun, S. Wu, W. H. Chen, L. P. Coelho, K. Ning, *Brief Bioinform* **2022**, *23* (6), <https://doi.org/10.1093/bib/bbac396>.

[24] A. Azhie, D. Sharma, P. Sheth, F. A. Qazi-Arisar, R. Zaya, M. Naghibzadeh, K. Duan, S. Fischer, K. Patel, C. Tsien, N. Selzner, L. Lilly, E. Jaeckel, W. Xu, M. Bhat, *Lancet Digit Health* **2023**, *5* (7), e458, <https://doi.org/10.1016/S2589-7500(23)00068-7>.

[25] D. L. X. Fung, X. Li, C. K. Leung, P. Hu, *Bioinform Adv* **2023**, *3* (1), vbad059, <https://doi.org/10.1093/bioadv/vbad059>.

[26] J. M. Choi, M. Ji, L. T. Watson, L. Zhang, *Bioinformatics* **2023**, *39* (5), <https://doi.org/10.1093/bioinformatics/btad286>.

[27] M. Bhat, M. Rabindranath, B. S. Chara, D. A. Simonetto, *J Hepatol* **2023**, *78* (6), 1216, <https://doi.org/10.1016/j.jhep.2023.01.006>.

[28] A. Zand, Z. Stokes, A. Sharma, W. K. van Deen, D. Hommes, *Dig Dis Sci* **2022**, *67* (10), 4874, <https://doi.org/10.1007/s10620-022-07506-8>.

[29] L. J. Marcos-Zambrano, K. Karaduzovic-Hadziabdic, T. Loncar Turukalo, P. Przymus, V. Trajkovik, O. Aasmets, M. Berland, A. Gruca, J. Hasic, K. Hron, T. Klammsteiner, M. Kolev, L. Lahti, M. B. Lopes, V. Moreno, I. Naskinova, E. Org, I. Paciência, G. Papoutsoglou, R. Shigdel, B. Stres, B. Vilne, M. Yousef, E. Zdravevski, I. Tsamardinos, E. Carrillo de Santa Pau, M. J. Claesson, I. Moreno-Indias, J. Truu, *Front Microbiol* **2021**, *12*, 634511, <https://doi.org/10.3389/fmicb.2021.634511>.

[30] K. D. Curry, M. G. Nute, T. J. Treangen, *Emerg Top Life Sci* **2021**, *5* (6), 815, <https://doi.org/10.1042/ETLS20210213>.

[31] I. S. Stafford, M. M. Gosink, E. Mossotto, S. Ennis, M. Hauben, *Inflamm Bowel Dis* **2022**, *28* (10), 1573, <https://doi.org/10.1093/ibd/izac115>.

[32] M. J. McGeachie, J. E. Sordillo, T. Gibson, G. M. Weinstock, Y. Y. Liu, D. R. Gold, S. T. Weiss, A. Litonjua, *Sci Rep* **2016**, *6*, 20359, <https://doi.org/10.1038/srep20359>.

[33] J. Lugo-Martinez, D. Ruiz-Perez, G. Narasimhan, Z. Bar-Joseph, *Microbiome* **2019**, *7* (1), 54, <https://doi.org/10.1186/s40168-019-0660-3>.

[34] R. Armoni, E. Borenstein, *Front Microbiol* **2022**, *13*, 909313, <https://doi.org/10.3389/fmicb.2022.909313>.

[35] M. Shahin, B. Ji, P. D. Dixit, *NPJ Syst Biol Appl* **2023**, *9* (1), 26, <https://doi.org/10.1038/s41540-023-00285-6>.

[36] K. D. Baksi, B. K. Kuntal, S. S. Mande, *Front Microbiol* **2018**, *9*, 36, <https://doi.org/10.3389/fmicb.2018.00036>.

[37] G. K. Gerber, A. B. Onderdonk, L. Bry, *PLoS Comput Biol* **2012**, *8* (8), e1002624, <https://doi.org/10.1371/journal.pcbi.1002624>.

[38] S. Kodikara, S. Ellul, K. A. Lê Cao, *Brief Bioinform* **2022**, *23* (4), <https://doi.org/10.1093/bib/bbac273>.

[39] P. Laccoureye, C. Bielza, P. Larranaga, Explainable machine learning of longitudinal multi-omics microbiome. MDPI: Mathematics, **2022**; Vol. 10.

[40] N. S. Seyed Tabib, M. Madgwick, P. Sudhakar, B. Verstockt, T. Korcsmaros, S. Vermeire, *Gut* **2020**, *69* (8), 1520, <https://doi.org/10.1136/gutjnl-2019-320065>.

[41] M. M. David, C. Tataru, Q. Pope, L. J. Baker, M. K. English, H. E. Epstein, A. Hammer, M. Kent, M. J. Sieler, R. S. Mueller, T. J. Sharpton, F. Tomas, R. Vega Thurber, X. Z. Fern, *mSystems* **2022**, *7* (1), e0105821, <https://doi.org/10.1128/msystems.01058-21>.

[42] P. Li, H. Luo, B. Ji, J. Nielsen, *Microb Cell Fact* **2022**, *21* (1), 241, <https://doi.org/10.1186/s12934-022-01973-4>.

[43] R. H. Medina, S. Kutuzova, K. N. Nielsen, J. Johansen, L. H. Hansen, M. Nielsen, S. Rasmussen, Machine learning and deep learning applications in microbiome research. Nature: ISME Communications, **2022**.
